# Modeling human papillomavirus and cervical cancer in the United States for analyses of screening and vaccination

**DOI:** 10.1186/1478-7954-5-11

**Published:** 2007-10-29

**Authors:** Jeremy D Goldhaber-Fiebert, Natasha K Stout, Jesse Ortendahl, Karen M Kuntz, Sue J Goldie, Joshua A Salomon

**Affiliations:** 1 Department of Health Policy and Management, Harvard School of Public Health, Boston, USA; 2 Program in Health Decision Science, Harvard School of Public Health, Boston, USA; 3 Departments of Health Policy and Management and Biostatistics, Harvard School of Public Health, Boston, USA; 4 Division of Health Policy and Management of the School of Public Health, University of Minnesota, Minneapolis, USA; 5 Department of Population and International Health, Harvard School of Public Health, Boston, USA; 6 Harvard University Initiative for Global Health, Cambridge, USA

## Abstract

**Background:**

To provide quantitative insight into current U.S. policy choices for cervical cancer prevention, we developed a model of human papillomavirus (HPV) and cervical cancer, explicitly incorporating uncertainty about the natural history of disease.

**Methods:**

We developed a stochastic microsimulation of cervical cancer that distinguishes different HPV types by their incidence, clearance, persistence, and progression. Input parameter sets were sampled randomly from uniform distributions, and simulations undertaken with each set. Through systematic reviews and formal data synthesis, we established multiple epidemiologic targets for model calibration, including age-specific prevalence of HPV by type, age-specific prevalence of cervical intraepithelial neoplasia (CIN), HPV type distribution within CIN and cancer, and age-specific cancer incidence. For each set of sampled input parameters, likelihood-based goodness-of-fit (GOF) scores were computed based on comparisons between model-predicted outcomes and calibration targets. Using 50 randomly resampled, good-fitting parameter sets, we assessed the external consistency and face validity of the model, comparing predicted screening outcomes to independent data. To illustrate the advantage of this approach in reflecting parameter uncertainty, we used the 50 sets to project the distribution of health outcomes in U.S. women under different cervical cancer prevention strategies.

**Results:**

Approximately 200 good-fitting parameter sets were identified from 1,000,000 simulated sets. Modeled screening outcomes were externally consistent with results from multiple independent data sources. Based on 50 good-fitting parameter sets, the expected reductions in lifetime risk of cancer with annual or biennial screening were 76% (range across 50 sets: 69–82%) and 69% (60–77%), respectively. The reduction from vaccination alone was 75%, although it ranged from 60% to 88%, reflecting considerable parameter uncertainty about the natural history of type-specific HPV infection. The uncertainty surrounding the model-predicted reduction in cervical cancer incidence narrowed substantially when vaccination was combined with every-5-year screening, with a mean reduction of 89% and range of 83% to 95%.

**Conclusion:**

We demonstrate an approach to parameterization, calibration and performance evaluation for a U.S. cervical cancer microsimulation model intended to provide qualitative and quantitative inputs into decisions that must be taken before long-term data on vaccination outcomes become available. This approach allows for a rigorous and comprehensive description of policy-relevant uncertainty about health outcomes under alternative cancer prevention strategies. The model provides a tool that can accommodate new information, and can be modified as needed, to iteratively assess the expected benefits, costs, and cost-effectiveness of different policies in the U.S.

## Background

In the United States, cervical cancer screening using repeated cervical cytology at frequent intervals has substantially reduced the incidence of invasive cancer, although there are still more than 3,000 deaths annually, and disparities in cancer outcomes persist [1-4]. With the development of reliable assays to detect high-risk, oncogenic types of human papillomavirus (HPV) and vaccines that are highly efficacious in preventing HPV types 16 and 18 in women not previously infected with these types, there are important questions to address with respect to cervical cancer prevention [5-8]. The two prophylactic vaccines that are currently in clinical trials include a quadrivalent vaccine that targets HPV-6/11/16/18 (Gardasil; Merck & Co., Inc., Whitehouse Station, New Jersey) and bivalent vaccine that targets HPV-16/18 (Cervarix; GlaxoSmithKline, Uxbridge, Middlesex, United Kingdom).

From both individual and population perspectives, options for primary and secondary prevention of cervical cancer would ideally be deployed synergistically to improve cancer outcomes, reduce disparities, minimize the risk of over-detection of abnormalities likely to resolve on their own, and enhance the cost-effectiveness of cervical cancer control. However, evaluating outcomes associated with different screening and vaccination strategies is challenging, since the interventions are applied at different time points and target different biologic processes along the spectrum of HPV infection, carcinogenesis, and invasive cancer. As such, no single, empirical study will be able to evaluate all possible strategies, and even studies aimed at assessing the benefits of one or two approaches would require extremely large sample sizes with extensive follow-up because of the long time course over which individuals are vulnerable to acquiring HPV infection and relatively low cancer incidence [6,9]. Integrating the best-available epidemiologic data, computer-based mathematical models used in a decision-analytic framework can identify those factors most likely to influence outcomes and can inform decisions that need to be made amidst considerable uncertainty.

Given the inevitable uncertainty around input parameters to any mathematical model, an important feature of many modeling efforts is the calibration of these parameter values in reference to observed epidemiologic data. Many previous cervical cancer models used in cost-effectiveness analyses have relied on manual calibration methods to fit to select sources of epidemiologic data [10-21], a reasonable approach for the questions addressed in previous analyses [22]. However, the increased availability of epidemiologic data – together with the complexities of comparing vaccination of adolescents against two high-risk HPV types and screening for high-grade cervical intraepithelial neoplasia (CIN) in women throughout much of adulthood – demands a more complex model with a more systematic approach to translating parameter uncertainty into the corresponding uncertainty around policy outcomes. Like others, our group has begun to apply more formal approaches to calibrating to multiple sources of data simultaneously [22]. Our objectives in this paper are to (1) describe a formal approach to the parameterization, calibration, and evaluation of a cervical cancer model for the U.S.; and (2) use this model to predict health outcomes, as well as the uncertainty surrounding these predictions, associated with cervical cancer screening, HPV-16/18 vaccination, and combinations of vaccination and screening.

## Methods

### Overview

We developed a microsimulation model of cervical carcinogenesis reflecting the best currently available evidence. The model development process included definition of model structure, parameterization, calibration, and evaluation of model performance. Parameter ranges for model inputs relating to natural history were defined from longitudinal cohort studies. Observed epidemiologic data on outcomes such as age-specific HPV prevalence and cervical cancer incidence prior to widespread screening were used to define targets for calibration of model parameters. Parameter values were sampled from the defined ranges, and simulations undertaken for each sampled parameter set. The goodness-of-fit (GOF) of modeled outputs resulting from each candidate set of input parameter values was evaluated using likelihood-based GOF scores calculated based on the full array of calibration targets. We identified a subset of the sampled parameter combinations that had scores that were statistically indistinguishable from that of the best-fitting set. We evaluated model performance in terms of consistency between modeled outcomes and those from large, population-based studies. The types and sources of data used in each of the three steps of model parameterization, calibration, and evaluation of performance are summarized in Table 1[14,20,21,23-102]. To assess the implications of our calibration approach in terms of the uncertainty in modeled outcomes that follows from empirically-calibrated input parameters, we examined the range of modeled cancer incidence reductions that could be expected across the calibrated parameter sets in simulations of screening (following current guidelines), widespread administration of an HPV vaccine, and a combination of vaccination and screening. Additional details of the model type, structure, parameterization, calibration, and evaluation are provided in the Appendix (see Additional File 1).

**Table 1:** Data sources used in model parameterization, calibration, and performance evaluation

**Modeling Step**	**Sources**
**Parameterization**	***HPV infection rates***
	*Primary Articles*: [23-27]
	*Reviews*: [14,20,21]
	***Progression rates to/within CIN***
	*Primary Articles*: [28-37]
	*Reviews*: [14,20,21,38,39]
	***Regression rates from CIN and HPV clearance rates***
	*Primary Articles*: [37,40,41]
	*Reviews*: [14,20,21,39]
	***Progression rates to/within cancer and cancer detection***
	*Reviews*: [14,20,21,42]
	***All-cause and cancer mortality rates***
	*Reviews*: [14,42,44]

**Calibration**	***Age-specific cervical cancer incidence***
	[43]
	***Cumulative cervical cancer incidence***
	[43,44]
	***Age-specific prevalence of high-risk and low-risk HPV***
	[45-61]
	***Age-specific prevalence of CIN1 and CIN2,3***
	[45,47,50,62-71]
	***HPV type distribution by CIN/cancer status***
	*Primary Articles*: [58,72-90]
	*Review*: [91-94]
	***Type-specific duration of HPV infections in younger and older women***
	[41]

Evaluation*	***Age-specific high-risk HPV prevalence and age-specific HSIL+ cytology prevalence***
	[95]
	***Proportion of CIN1 and CIN2,3 that are HPV-16 and HPV-18 or other high-risk HPV infected***
	[96]
	***Screening reduction in age-specific cancer incidence rates and cancer stage at detection***
	[43,97]

* Screening patterns used in model evaluation: [98-102]

### Model structure, parameterization, and simulation

**Figure 1:**
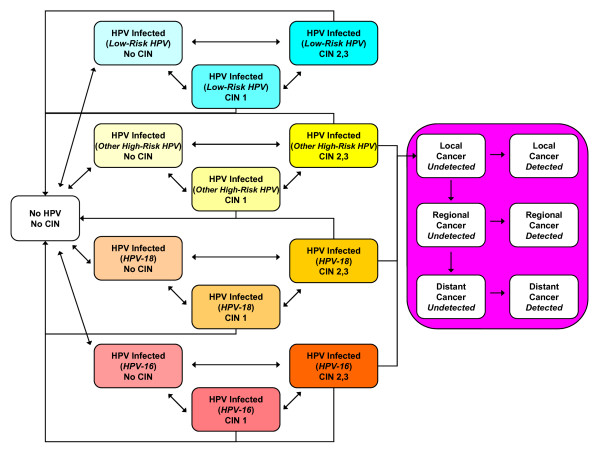
**Model natural history schematic**. Each ellipse represents a state in the natural history model. HPV is stratified by type. Each month, a woman has a chance of transitioning from her current state along one of the arrows emanating from that state to another state or else staying in her current state. All women also have a chance of dying from all-cause mortality, and women with invasive cancer have an additional stage-specific chance of dying from their cancer.

The stochastic microsimulation model simulates the transitions of individuals between a set of mutually exclusive health states (Figure 1). In the model, HPV infection is stratified into 5 categories: not infected; HPV-16; HPV-18; other high-risk types (category includes types 31, 33, 35, 39, 45, 51, 52, 56, 58, 59, and 68); and low-risk types. CIN status is modeled in three categories: no CIN; CIN 1, and CIN2,3. Individual females enter the model at age 9 prior to sexual debut and remain in the model for the entirety of their lives. Transitions between health states occur at monthly intervals and depend on HPV type, age, history of prior HPV infection, type-specific natural immunity, previous treatment for CIN, and screening patterns. Each month, a woman has an age- and type-specific probability of being infected with HPV. Modeled probabilities of age-related HPV infection act as a proxy for the probability of being sexually active combined with the probability of transmission and distribution of HPV types among sexual partners. The model also has the capability of considering the indirect effects on health outcomes associated with herd immunity by modifying incidence rates based on output from a dynamic model [103]. Most women with HPV infections will develop transient abnormalities reflecting productive HPV infection, and some will progress to CIN2,3. Women infected with high-risk HPV types and having persistent high-grade CIN may progress to invasive cancer, and those with invasive cancer can develop symptoms or progress to the next stage of cancer. We assume that symptomatic women with invasive cancer receive stage-specific treatment for their disease and are subject to the corresponding stage-specific survival rates. From every health state and in every month, women face competing mortality risks from all other causes.

We derived initial estimates and ranges for monthly transition probabilities required by the model from published literature (Table 2). Because of uncertainty about the estimated quantities within each study and heterogeneity across studies based on methodological and population differences, the upper and lower bound of each search range were defined to be broadly inclusive of (1) study-reported confidence intervals when available; (2) the highest and lowest estimate reported from different data sources; and (3) expert opinion.

**Table 2:** Model natural history parameters, search ranges, and calibrated parameters*

**Variables ** †	**Before Calibration**		**Calibration Results**	
			**Parameter Ranges**	
	**Parameter Inputs**	**Parameter Multiplier** **Search Ranges**	**Minimum**	**Maximum**
**Progression**				

**From Normal to HPV Infection ‡**				
**- Low-Risk HPV**	0.1 – 34.5	1.0 – 4.0	0.2	137.3
**- High-Risk HPV-16**	0.1 – 23.4	1.0 – 8.0 §§	0.5	188.6
**- High-Risk HPV-18**	0.0 – 13.8	1.0 – 8.0 §§	0.0	110.1
**- Other High-Risk HPV**	1.2 – 114.5	1.0 – 8.0	1.5	937.3
**From HPV Infection to CIN1 §**				
**- Low-Risk HPV**	55.8 – 64.7	0.1 – 6.0	51.7	377.5
**- High-Risk HPV-16**	57.5 – 102.3	0.1 – 6.0	13.2	621.0
**- High-Risk HPV-18**	57.5 – 102.3	0.1 – 6.0	14.8	595.7
**- Other High-Risk HPV**	57.5 – 102.3	0.1 – 6.0	27.3	615.7
**From HPV Infection to CIN2,3 ¶**				
**- Low-Risk HPV**	0.4 – 9.3	0.0 – 0.1	0.0	0.9
**- High-Risk HPV-16**	2.2 – 46.7	0.1 – 1.0	0.3	46.6
**- High-Risk HPV-18**	2.2 – 46.7	0.0 – 0.1	0.0	4.4
**- Other High-Risk HPV**	2.2 – 46.7	0.0 – 0.1	0.0	4.6
**From CIN1 to CIN2,3**				
**- Low-Risk HPV**	0.4 – 9.3	0.5 – 4.0	0.3	37.0
**- High-Risk HPV-16**	2.2 – 46.7	0.5 – 6.0	1.2	282.4
**- High-Risk HPV-18**	2.2 – 46.7	0.1 – 4.0	0.8	185.1
**- Other High-Risk HPV**	2.2 – 46.7	0.1 – 4.0	0.4	115.1
**From CIN2,3 to Local Cancer ||**				
**- Low-Risk HPV**	0.0		0.0	0.0
**- High-Risk HPV-16**	0.6 – 72.2	1.0 – 5.0	1.1	362.0
**- High-Risk HPV-18**	0.6 – 72.2	1.0 – 5.0	0.8	362.3
**- Other High-Risk HPV**	0.6 – 72.2	1.0 – 3.0	0.7	216.1
**Progression within Cancer**				
**- Local Cancer to Regional Cancer**	242.4		242.4	242.4
**- Regional Cancer to Distant Cancer**	303.8		303.8	303.8

**Regression**				

From CIN2,3 to Normal **				
**- Low-Risk HPV**	11.8 – 42.4	0.5 – 5.0	6.4	201.3
**- High-Risk HPV-16**	11.8 – 42.4	0.5 – 5.0	6.5	197.8
**- High-Risk HPV-18**	11.8 – 42.4	0.5 – 5.0	6.5	212.0
**- Other High-Risk HPV**	11.8 – 42.4	0.5 – 5.0	6.3	213.2
**From CIN1 to Normal ††**				
**- Low-Risk HPV**	371.7	0.5 – 5.0	229.8	1968.9
**- High-Risk HPV-16**	371.7	0.5 – 5.0	193.2	1948.8
**- High-Risk HPV-18**	371.7	0.5 – 5.0 §§	193.2	1948.8
**- Other High-Risk HPV**	371.7	0.5 – 5.0	310.3	1974.0
**From HPV Infection to Normal ††**				
**- Low-Risk HPV**	371.7	1.5 – 6.0	1,114.2	2415
**- High-Risk HPV-16**	371.7	1.5 – 6.0	589.7	2324.4
**- High-Risk HPV-18**	371.7	1.5 – 6.0 §§	589.7	2324.4
**- Other High-Risk HPV**	371.7	1.5 – 6.0	778.1	2355.7

**Cancer detected by symptoms**				

**- Local Cancer**	210.6		210.6	210.6
**- Regional Cancer**	916.1		916.1	916.1
**- Distant Cancer**	2302.6		2302.6	2302.6

**Mortality**				

**- All Cause**	0.1 – 297.1		0.1	297.1
**- Local Cancer**	19.2		19.2	19.2
**- Regional Cancer**	140.0		140.0	140.0
**- Distant Cancer**	489.9		489.9	489.9

**Natural Immunity ‡‡**				

**- Low-Risk HPV**	0%		0%	0%
**- Other High-Risk HPV**	100%	0.0 – 1.0	4%	90%
**- High-Risk HPV-16**	100%	0.0 – 1.0 §§	40%	99%
**- High-Risk HPV-18**	100%	0.0 – 1.0 §§	26%	100%

* HPV = human papillomavirus; CIN = cervical intraepithelial neoplasia. Values are expressed as yearly rates per 1,000 women, unless otherwise noted.

† Ranges represent age-specific values. Ranges for calibrated values represent the combination multipliers applied to a pre-calibration value or pre-calibration age-specific values, where appropriate.

‡ Multipliers were constrained to be higher for HPV-16 and HPV-18 than for other high-risk HPV.

§ Although pre-calibration rates of progression and the range of multipliers were consistent among all high-risk HPV types, the multipliers were allowed to vary independently by type in the parameter searches.

¶ A proportion of women with HPV transition directly to CIN2,3.

**|| **Infection with high-risk HPV is considered necessary for progression to invasive cancer

** 70% of women with CIN2,3 clear their infection, 15% retain detectable HPV infection with CIN1, and 15% retain HPV infection without any CIN.

†† Although pre-calibration rates of regression and the range of multipliers were consistent among all HPV types, the multipliers were allowed to vary independently by type in the parameter searches.

‡‡ Immunity represents the percentage reduction in risk of subsequent, type-specific infections after a woman has cleared an infection with the same type. Immunity for HPV-16 and HPV-18 are constrained to be higher than immunity for the category of other high-risk types.

§§ Search range conditional on value randomly drawn for another parameter. Regression rates for HPV-16 and HPV-18 associated CIN1 are assumed to be equal. Clearance rates for HPV-16 and HPV-18 are assumed to be equal. Natural immunity for HPV-16 and HPV-18 are both constrained to be higher than immunity to the category of other high-risk types.

### Model calibration

Our approach to model calibration was based on a multi-dimensional random search in which input parameters values were sampled from plausible ranges defined as described above, and then separate simulations of the model were run using each set of sampled parameter values.

#### Calibration targets

We defined 84 epidemiologic outcomes that comprised targets for calibration, within the following categories: type- and age-specific prevalence of HPV; type- and age-specific duration of HPV infection; age-specific prevalence of CIN1 and CIN2,3; age-specific cancer incidence; lifetime cancer risk; and HPV type-distribution within CIN and cancer. For each calibration target, we determined a 95% confidence interval using data arising from population-based studies [104]. When multiple data sources were available, we used random-effects models for data synthesis to produce combined point estimates and confidence intervals [105]. For some calibration targets, for which the quantities of interest were expected to vary across epidemiologic settings, we limited the range of data sources that informed the definition of our targets. For example, targets on HPV and CIN prevalence were based only on North American studies, despite availability of a wider range of possible data sources. Because the model is intended for policy analyses of screening and vaccination in the United States, targets relating to cancer were based exclusively on data from the U.S.

To establish calibration targets for *age-specific prevalence of HPV and CIN *we included studies that provided sufficient information on sample size and prevalence of age-specific infection with high-risk or low-risk HPV types [45-61]. Similarly, we included only studies that provided sufficient information on sample size and prevalence of CIN1 or CIN2,3 [45,47,50,62-71]. For targets relating to *duration of infection*, data were derived from a single, longitudinal Brazilian study that collected frequent, repeated measures of HPV status in a large cohort of women over an average follow-up of 53 months [41], supplemented with secondary data from studies with shorter periods of observation from the U.S.

To calibrate model parameters governing the natural history of disease in the absence of screening, targets on the *age-specific incidence of invasive cervical cancer *were defined based on 1959–60 data reported from multiple U.S. registries to the International Agency for Research on Cancer (IARC) [43]. Observations from each registry were treated as outcomes of independent experiments and combined using a random-effects model [105]. To define targets on the *lifetime risk of cancer*, we incorporated the lower and upper confidence intervals from the age-specific cancer incidence rates and all-cause, age-specific U.S. mortality rates in a multiple-decrement, life table approach to account for competing mortality hazards [44,106,107].

We defined targets on *HPV type distribution in CIN and cancer *based primarily on the systematic reviews of Clifford et al., supplemented by subsequently published studies [58,72-94]. Specifically, we estimated the proportion of patients with CIN2,3 infected with HPV-16, HPV-18, or another high-risk HPV type; the proportion of patients with CIN1 infected with HPV-16/18 or another high-risk type; and the proportion of patients with invasive cervical cancers infected with either HPV-16 or HPV-18.

#### Parameter set selection

For model inputs, we used age-specific estimates derived from longitudinal studies to define the baseline shape of each parameter curve with respect to age, but searched plausible ranges around these inputs by specifying ranges for scalar multipliers applied to these curves. The plausible ranges around input parameter values comprised a joint uniform prior distribution for the inputs. We randomly sampled 1,000,000 parameter sets from this joint prior distribution.

For each set of sampled parameter values, we simulated a population of 100,000 individuals in the model and then compared the modeled outputs corresponding to each of the 84 calibration targets to the target values for these outputs. The formal comparison was based on a likelihood score, computed under the assumption that calibration targets were characterized by independent, normal probability density functions, with means and standard deviations derived from the empirical 95% confidence intervals. An overall goodness-of-fit (GOF) score was computed as negative two times the sum of the log-likelihood scores for each target. To compare the fit of different parameter sets, we assumed that the distribution of GOF scores across simulations may be approximated by a chi-square distribution with the number of degrees of freedom equal to the number of calibration targets.

With likelihood-based GOF scores, we identified multiple parameter set combinations whose outputs were simultaneously consistent with calibration targets derived from epidemiologic data. In addition to this first criterion of overall goodness-of-fit, we also evaluated candidate parameter sets based on a second criterion, of goodness-of-fit with respect to a subset of high-priority targets, based on their relevance to policy questions regarding vaccination and screening. First, we determined our best-fitting parameter set as the one with the lowest overall GOF score – the model-generated input parameter set whose simulated outputs were simultaneously closest to all calibration targets. We identified those parameter sets with GOF scores that were statistically indistinguishable from the GOF score of the best-fitting set (based on a likelihood ratio test with p < 0.05), and considered these to be good-fitting also. To further restrict the selection of good-fitting parameter values to those that provided the best fit to high-priority targets, we computed a GOF subscore based on the following high-priority targets: prevalence of CIN1 at ages 25–29 and 35–39 years, proportion of HPV-16/18 in CIN1, proportion of HPV-16 in CIN2,3 and cancer, proportion of HPV-18 in cancer, and cancer incidence at ages 45–49, 55–59, and 70–74 years. Finally, we accepted those parameter sets that were good-fitting and had high-priority GOF subscores amongst the top 1% of all parameter sets.

For efficiency, subsequent analyses were based on a random resample of 50 parameter sets from the array of accepted parameter sets to preserve the representation of overall parameter uncertainty while reducing the computational intensity required for the simulations.

### Model evaluation

We evaluated the performance of the model in terms of its external consistency and face validity by comparing model output to data from several large U.S. screening studies not used in the parameterization or calibration of the natural history model. These included the ASCUS/LSIL Triage Study for Cervical Cancer (ALTS) and Portland Kaiser Permanente studies as well as data from the NCI's Surveillance Epidemiology and End Results (SEER) Program [95-97]. For all evaluation targets, we derived both point estimates and 95% confidence intervals from the empirical data.

Baseline data from the Portland Kaiser Permanente study were used to calculate the cross-sectional, age-specific prevalence of high-risk HPV and of cervical cytology results that were high-grade squamous intraepithelial lesion or worse (HSIL+). Baseline data from the ALTS study were used to calculate the cross-sectional proportions of HPV-positive persons with CIN1 and CIN2+ (e.g., histology of CIN2 or worse) having HPV-16, HPV-18, or other high-risk HPV types. Data from SEER for 2003 were used to derive incidence of age-specific invasive cervical cancer detected in the presence of cervical cancer screening, and analogous estimates were calculated from IARC data for the U.S. prior to widespread screening. We estimated the incidence reductions due to screening by subtracting SEER incidence rates from IARC rates by age. SEER data from 1996 to 2000 were used to calculate the proportion of invasive cervical cancer cases detected at each SEER historical stage (local, regional, and distant).

To compare consistency of model results to our external evaluation targets, we simulated 5 screening scenarios for each of the 50 parameter sets from the calibrated natural history model. These included no screening and screening using cervical cytology at 4 levels of intensity: every 1, 2, 3, or 5 years from ages 18 to 70. Additional assumptions about screening, diagnosis, and treatment are provided in the Appendix (see Additional file 1). Model outputs for these simulations were based on cohorts of 1,000,000 women.

Because data from these studies were derived from women with different past patterns of screening, we combined modeled outputs from our 5 screening scenarios to produce modeled outputs that would be consistent with a cohort of women whose cervical cancer screening patterns matched nationally observed, age-specific patterns of screening [98,102]. For the ALTS targets, age-specific modeled outputs were then collapsed across age categories using a weighted average based upon the age structure of the ALTS data. Since SEER stage at cancer detection targets were based on women with detected cervical cancer, matched modeled outputs (e.g., the number of cervical cancer cases detected at each stage for each screening scenario) were combined using screening patterns derived from case-control studies of women with diagnosed cervical cancer [99-101]. We assessed model performance in terms of its external consistency and face validity using a benchmark of overlap between the model range and the study's confidence interval or range. Similarity in age patterns between modeled and study outcomes based on visual inspection was used as a further criterion for assessing model performance.

### Quantifying the impact of model calibration

A key motivation of this study was to better reflect parameter uncertainty critical to policy analyses, for example, evaluations of cost-effectiveness of vaccination and screening interventions to prevent cervical cancer. While a full consideration of alternative prevention strategies is beyond the scope of this study, we provide an illustrative example by using the empirically calibrated model to project the reduction in cervical cancer that would be expected with HPV vaccination and cytology-based screening at different frequencies. We assumed the prophylactic HPV vaccine would be given to all women prior to age 12, and assumed complete, lifelong protection against HPV-16 and -18. For each of the 50 resampled good-fitting parameter sets, we estimated cumulative cervical cancer incidence with and without vaccination or screening and examined the distribution across parameter sets, of reductions in cervical cancer incidence, expressed as percentages. The distributions of cancer incidence reduction under alternative prevention strategies illustrate how calibration of model input parameters to epidemiologic data can be used to quantify the uncertainty around results of policy interest.

Across the 50 good-fitting parameter sets, we calculated the pair-wise correlation coefficients for expected cervical cancer reduction due to screening at different frequencies, vaccination without screening, and screening at different frequencies and vaccination used in combination. For this analysis, the unit of observation was the parameter set. The significance (p < 0.05) of the correlation was assessed using a Bonferroni correction for multiple comparisons. The aim of the analysis was to evaluate relationship of benefit derived from screening and vaccination used alone or in combination: specifically, whether parameter sets in which screening produced more benefit also showed more benefit from vaccination and whether the benefit from screening used at different frequencies combined with vaccination was correlated within parameter sets.

### Statistical analysis

The microsimulation was implemented in C++ on high-performance Linux computer clusters. Analyses of results were performed with Stata/SE 9.2 for Windows (StataCorp LP, College Station, TX) and Microsoft Office Excel 2003 SP2 (Microsoft Corporation, Redmond, WA).

## Results

### Model calibration

For 1,000,000 randomly generated input parameter sets, approximately 1,000 input parameter sets had GOF scores that were statistically indistinguishable from that of the best-fitting set (at p < 0.05). Of these input parameter sets, 183 met the additional criterion of being in the top 1 percent of fits for the high-priority subset of calibration targets. From the parameter sets accepted based on these two criteria, 50 were randomly resampled for further analyses and evaluation. Table 2 shows the results of calibration on the range of model input parameters. Further details on the results on calibration are documented in the Appendix (see Additional file 1).

**Figure 2:**
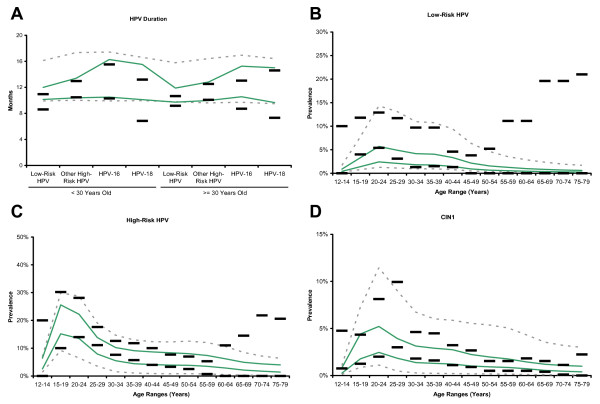
**Calibration to empirical data**. (Panels A through D) Black horizontal bars represent the upper and lower bounds of the 95% confidence intervals of each calibration target. Dashed gray lines represent model outputs prior to calibration and selection. Green lines represent model outputs after calibration and selection. Vertical axes represent duration, prevalence, proportion, or incidence rate as appropriate, and horizontal axes represent age or other categories as appropriate.

**Figure 3:**
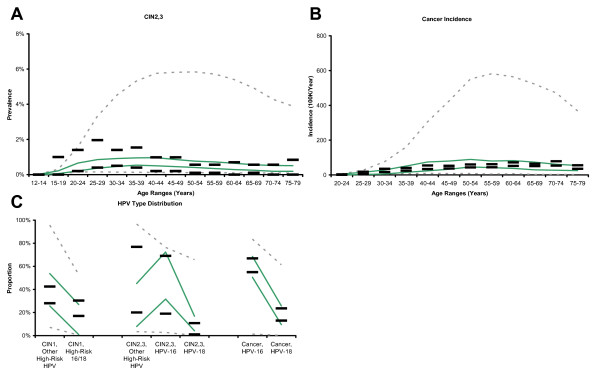
**Calibration to empirical data**. (Panels A through C) Black horizontal bars represent the upper and lower bounds of the 95% confidence intervals of each calibration target. Dashed gray lines represent model outputs prior to calibration and selection. Green lines represent model outputs after calibration and selection. Vertical axes represent duration, prevalence, proportion, or incidence rate as appropriate, and horizontal axes represent age or other categories as appropriate.

Figure 2 and Figure 3 compare the calibration targets to the range of model outputs before and after calibration. The process of calibration improved model fit for all calibration targets. The greatest improvement in model fit is noted in age-specific prevalence of CIN2,3 and age-specific incidence of invasive cervical cancer. Observed durations for low-risk HPV in all ages, for HPV-16 in women younger than 30 years, and for HPV-18 in women 30 years or older are overestimated for some parameter sets, but the pattern of the increased duration for higher risk HPV types within age categories is consistent with calibration targets. Age-specific low-risk HPV prevalence is generally low for women under 30, and CIN1 prevalence is low for 25–29 year-olds. HPV type distribution within neoplasia and cancer category, age-specific prevalence of high-risk HPV and CIN2,3, and age-specific incidence of invasive cancer demonstrate consistency between modeled outcomes and calibration targets.

### Model evaluation

**Figure 4:**
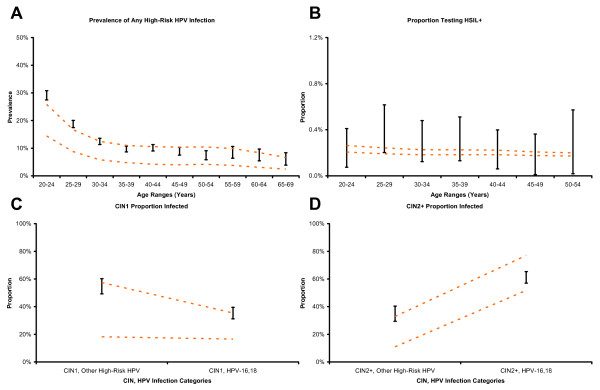
**External consistency of model output compared to independent data**. (Panels A through D) Black vertical bars represent the 95% confidence intervals of each evaluation target. Dashed orange lines represent the results from matched model outputs in the presence of screening. Vertical axes represent prevalence, proportion, or incidence reduction as appropriate, and horizontal axes represent age or other categories as appropriate.

We compared the model-predicted screening outcomes across the spectrum of HPV infection and cervical cancer to selected studies not used in parameterization or calibration of the model. Figure 4A shows the 95% confidence intervals for age-specific high-risk HPV prevalence among women enrolled in the Portland Kaiser Permanente study compared with modeled outputs for women whose screening intensities were derived from national averages. Model output is consistent with the general shape of decline in high-risk HPV prevalence for older ages. For some parameterizations, the model produces lower estimates of age-specific prevalence than those from the Kaiser sample. Figure 4B shows the 95% confidence intervals for age-specific proportion of cytology results that were HSIL or worse among women enrolled in the Portland Kaiser Permanente study compared with modeled outputs for women whose screening intensities were derived from national averages. Modeled outputs fall within their respective confidence intervals for all age ranges.

Figure 4C and 4D show the 95% confidence intervals for the proportions of CIN1 and CIN2+ infected with either HPV-16 or HPV-18, or only with other high-risk HPV types, compared with modeled outputs for women whose screening intensities were derived from national averages. Model output is consistent with the general trend of an increasing proportion of neoplasia being infected with HPV-16 or HPV-18 as the severity of neoplasia increases. Although modeled ranges overlap the 95% confidence intervals of the ALTS study, they are wider and more variable for CIN1. For CIN2+, model output is more consistent with the study data.

**Figure 5:**
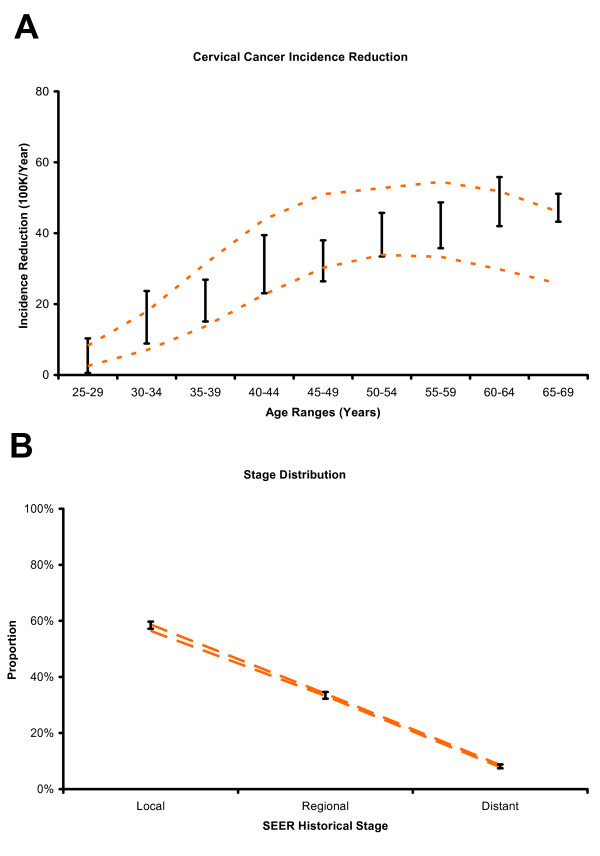
**External consistency of model output compared to independent data**. (Panels A and B) Black vertical bars represent the 95% confidence intervals of each evaluation target. Dashed orange lines represent the results from matched model outputs in the presence of screening. Vertical axes represent prevalence, proportion, or incidence reduction as appropriate, and horizontal axes represent age or other categories as appropriate.

Figure 5A shows the range of differences between observed age-specific detected cervical cancer incidence in the presence or absence of screening (from SEER 2003 and IARC 1959–1960, respectively) compared with the modeled outputs for women without screening and those women whose screening intensities were derived from national averages. Ranges from observed and modeled data overlap for all age categories. Even though the modeled ranges overlap for women above 60 years of age, the modeled reduction in age-specific detected cancer incidence tends to be lower than observed values for this age group. Figure 5B shows a comparison of modeled distribution of stage at detection among women with detected invasive cervical cancer to matched SEER data (1996–2000). Model output is consistent with the SEER cancer stage data.

### Impact of uncertainty

**Figure 6:**
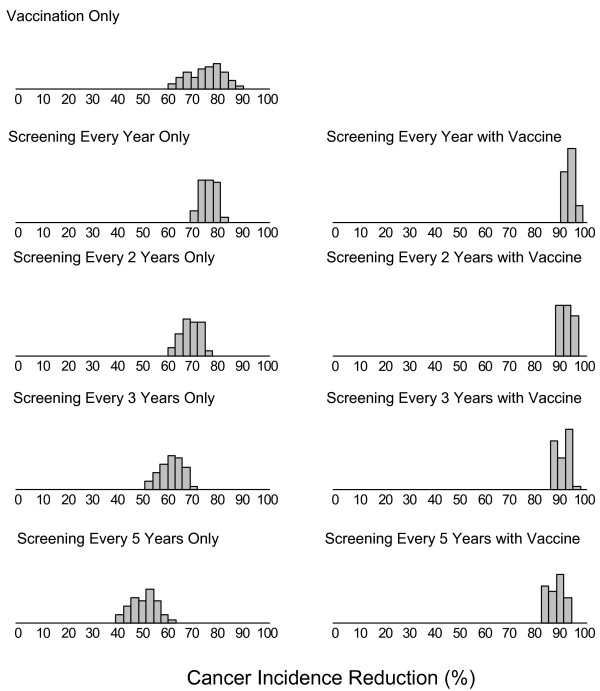
**Uncertainty in cancer reduction from alternative prevention strategies**. The figure depicts histograms (gray bars) representing the distribution of cancer reductions (x-axes) expected from HPV vaccination, cytology screening at 1, 2, 3, or 5 year intervals, and the combination of screening and vaccination. The distribution of cancer reduction represents the uncertainty in policy-relevant outcomes attributable to parameter uncertainty identified through calibration.

Figure 6 summarizes the distribution across 50 good-fitting parameter sets of cumulative cervical cancer incidence reductions with an HPV-16/18 vaccine, with current screening guidelines carried out at different frequencies, and with combined vaccination and screening. Reductions range from 60 to 88% for HPV vaccination. While the expected reduction in cancer incidence is 75%, parameter uncertainty implies a substantial range of possible benefits: more than 20% of the parameter sets yield reductions in incidence that differ from the mean reduction by more than 10 percentage points. Screening reduces cancer by 76% (range across all 50 parameter sets: 69–82), 69% (60–77), 62% (51–71), and 50% (39–61) for frequencies of every 1, 2, 3, and 5 years, respectively. Model results under frequent screening are less susceptible to parameter uncertainty than less frequent screening or vaccination alone. The range of cancer reduction from screening more frequently than every 3 years exhibits substantial overlap with vaccination. When screening and vaccination are combined cancer incidence reductions are 94% (90–97), 93% (89–97), 91% (86–96), and 89% (83–95) for frequencies of every 1, 2, 3, and 5 years, respectively. Uncertainty is further reduced under combined screening and vaccination programs, and less frequent screening combined with vaccination has expected benefits greater than vaccination alone or yearly screening.

Across parameter sets, estimated cancer reductions associated with screening at different frequencies are significantly positively correlated (Table 3); in other words, those parameter sets yielding high benefits for a given screening frequency also typically indicate high benefits for other screening frequencies as well. This high correlation suggests that factors that influence the effectiveness of screening programs at a variety of screening frequencies (e.g., duration in CIN2,3 prior to progressing to invasive cancer or the proportion of HPV infections of different types leading to CIN) are captured within parameter sets. There is no significant correlation between cancer reductions due to vaccination alone and those due to screening alone at any frequency. While screening strategies achieve benefit by detecting and removing CINs, some of which may have progressed to cancer, vaccination acts to prevent type-specific HPV infection. The pattern of correlation observed is consistent with the observation that uncertainty about HPV infection and clearance is loosely connected to CIN progression and regression. More importantly, vaccination combined with screening provides positive synergy for cancer prevention as evidenced by earlier results that screening alone and vaccination alone both produce substantial cancer reduction, screening alone and vaccination alone are not correlated, and the positive, significant correlation in cancer reduction as screening frequency is increased in the presence of vaccination.

**Table 3:** Pair-wise Correlations between Cancer Reductions due to Vaccination and Screening at Different Frequencies †

	Screen only, q1	Screen only, q2	Screen only, q3	Screen only, q5	Vaccine only	Screen and vaccine, q1	Screen and vaccine, q2	Screen and vaccine, q3	Screen and vaccine, q5
Screen only, q1	1.000								
Screen only, q2	0.981*	1.000							
Screen only, q3	0.968*	0.995*	1.000						
Screen only, q5	0.952*	0.985*	0.996*	1.000					
Vaccine Only	-0.043	-0.036	-0.044	-0.037	1.000				
Screen And Vaccine, q1	0.122	0.104	0.097	0.108	0.866*	1.000			
Screen And Vaccine, q2	0.143	0.129	0.125	0.138	0.848*	0.991*	1.000		
Screen And Vaccine, q3	0.139	0.124	0.121	0.137	0.845*	0.985*	0.998*	1.000	
Screen And Vaccine, q5	0.131	0.121	0.119	0.135	0.867*	0.984*	0.996*	0.997*	1.000

† q1, q2, q3, and q5 mean screening every 1, 2, 3, and 5 years respectively.

* Pair-wise correlation coefficient is significant (p < 0.05)

## Discussion

We systematically calibrated a microsimulation model of HPV and cervical cancer in the United States to multiple epidemiologic studies using a likelihood-based approach. By characterizing uncertainty in disease natural history using a model that includes both vaccination and screening capabilities, we demonstrated the impact of the approach in terms of quantifying the uncertainty about one policy-relevant outcome, the reduction of cervical cancer incidence.

Concordance between modeled age- and HPV type-specific prevalence outcomes was reasonable, with consistent fits achieved for important calibration targets including high-risk HPV prevalence, HPV type distribution within neoplasia and cancer, and cervical cancer incidence. In terms of external consistency, model performance was deemed reasonable by comparison of additional modeled outcomes in the presence of screening to independent studies not used to parameterize the model.

Prior research on model calibration and validation and the quantification of uncertainty has spanned many disease areas and disciplines. Important aspects of this research include data synthesis useful for model parameterization, calibration, and evaluation [105,108-110]; methods to use evidence in model calibration and quantification of parameter uncertainty [111-113]; and methods to alleviate the computational burden associated with all parts of model calibration and validation [114-118].

A broad range of modeling techniques has been used to evaluate cervical cancer screening and vaccination, though researchers have only recently begun to integrate, apply, and extend work on model calibration and validation techniques to models of HPV and cervical cancer [22]. Numerous computer models based on Markov cohorts have evaluated alternative cervical screening policies in the absence of HPV vaccination [10-21]. Additionally a European microsimulation of cervical cancer has been developed [119,120]. Subsequent models considered both screening and vaccination [121-125].

Dynamic transmission models have been reported for Finland [126,127] and the United States [103,128-130]. Key features of dynamic transmission models, which will become more important as data become available on vaccine efficacy in males, include their ability to incorporate the natural history of HPV in men and women, reflect transmission dynamics over time, and endogenously capture herd immunity. Because such models require data on HPV transmission and sexual partnerships data in addition to data on disease natural history, they involve more uncertain parameters, and thus far, have not considered oncogenic HPV types other than HPV-16 and HPV-18.

Several recent studies have undertaken methodological efforts related to those in this study. In a model of HPV in Brazil, Kim et al. [22] used an iterative approach to calibrate a natural history model of cervical cancer to longitudinal data to elucidate the differential impact of selected uncertain assumptions and then employed likelihood-based calibration. Based on data from a Canadian study, Burchell et al. [131] explored heterosexual HPV transmission probabilities, using a stochastic computer simulation to search for transmission probabilities consistent with the study's 95% confidence intervals. The model identified parameter uncertainty for transmission parameters in comparison to one epidemiologic study. Van de Velde et al. [125] calibrated a model of HPV and cervical cancer, identifying multiple parameter sets that fit North American epidemiologic data, including Canadian cancer data. However, their evidence combination methods did not account for differences in study size, and their fitting procedure was not likelihood-based. Like Van de Velde and colleagues, our model's unknown and uncertain inputs are calibrated to observed data, and multiple parameterizations are identified. Like Burchell et al., we identify plausible parameter combinations, relying on a likelihood-based approach to fit model outputs to confidence intervals derived from multiple data sources. Because our model simulates individual women and is analyzed as a first-order Monte Carlo simulation, in addition to capturing the effects of vaccination, it can account for each woman's previous history and allow her history of screening, vaccination, health, and behavior to affect her future risk.

Our study has a number of limitations. While we searched 1,000,000 parameter sets, searching more extensively might yield marginal improvements in fit to the observed data. On the other hand, the data used for calibration come from heterogeneous sources whose evidence is not entirely consistent. Insistence on an exact match to available data may lead to over-fitting and thereby underestimate uncertainty in model outcomes. Another limitation is the availability of data for model design and parameterization. Areas of particular importance include sexual activity patterns and HPV transmission in adolescents, characteristics of natural immunity, and host genetic heterogeneity. Results from our study support the need for further studies of HPV natural history in order to narrow parameter uncertainty. Findings from future studies will likely necessitate updating our current model and parameter estimates through recalibration. While our model can be linked indirectly to a separate dynamic transmission model, it does not directly capture herd immunity effects [103]; we opt instead for a detailed monthly microsimulation that includes multiple HPV types and allows for individual differences in risks and screening patterns based on past history – features that are less readily incorporated into standard dynamic transmission models. Finally, we recognize that a focus on cervical cancer omits the impacts of preventing other rarer cancers that occur in both men and women [132]. This is an important area of future work as better data become available.

Our model makes a set of causal assumptions about the natural history of HPV and cervical cancer, embodied in the model's structure, which are consistent with current biologic understanding of cervical disease. Systematic model calibration results in multiple alternative model parameterizations consistent with epidemiologic data. With our calibrated model, we were able to generate predictions about the effects of screening patterns in the U.S. on HPV, CIN, and cancer that were reasonably consistent with multiple independent studies. The model is not, however, intended as a formal model of causal effect that proves specific relationships in the biology and epidemiology of HPV and cervical cancer. Rather, the model is intended to comment on current questions of policy relevance from a decision analytic standpoint, reflecting the uncertainty in predicted outcomes that exists even in models that are consistent with observed population data.

While we report reasonably favorable model performance results with respect to three large studies not used in parameterization or calibration, model fits to sometimes wide confidence intervals are imperfect. One important future direction would be to continue to refine and expand the set of evaluation targets. When newer data from ongoing population studies of HPV vaccines emerge, they may provide useful opportunities to conduct evaluation exercises with respect to possible changes in the HPV type distribution between unvaccinated and vaccinated cohorts [133]. Another future direction is the assessment of model structure uncertainty. Research in other disease areas has relied largely on review and comparison of cost-effectiveness results to assess the differential effects of model structure assumptions [134-136]. Recent studies have compared alternative model structures developed by different groups [137,138]. Few studies in health-related areas have simultaneously considered model structure and parameter uncertainty [139,140].

## Conclusion

Our systematic approach to the parameterization, calibration, and evaluation of a complex microsimulation of HPV and cervical cancer identified many independent natural history parameter sets that fit equally well to multiple epidemiologic targets. As cervical cancer prevention options evolve, and as new evidence becomes available from ongoing studies, an empirically calibrated model is one of many tools that can provide policy-makers with important information on the expected benefits associated with different policies. By conducting comparative analyses of different strategies using a random sample of the good-fitting parameter sets, decision makers are also provided with a description of the uncertainty in policy outcomes that follows from uncertainty in model parameters.

## Abbreviations

ALTS – ASCUS/LSIL Triage Study for Cervical Cancer

ASCUS – Atypical squamous cells of unknown significance

CIN – Cervical intraepithelial neoplasia

DNA – Deoxyribonucleic acid

GOF – goodness-of-fit

HPV – Human papillomavirus

HSIL – High-grade squamous intraepithelial lesion

IARC – International Agency for Research on Cancer

LSIL – Low-grade squamous intraepithelial lesion

NCI – National Cancer Institute (United States)

SEER – Surveillance Epidemiology and End Results

US – United States of America

## Competing interests

The author(s) declare that they have no competing interests.

## Authors' contributions

JGF, NKS, KMK, SJG, and JAS participated in the conception and design of the study. JGF, KMK, and SJG participated in the acquisition of the data. All authors participated in the analysis and interpretation of the data. JGF drafted the initial manuscript. NKS, JO, KMK, SJG, and JAS critically reviewed manuscript and contributed important intellectual content. All authors read and approved the final manuscript.

## Supplementary Material

Additional file 1 An individual-based stochastic microsimulation of human papillomavirus and cervical cancer in the United States: Supplemental technical information. The appendix provided includes a supplementary description of the model structure, parameterization, calibration, and evaluation as well as information on the results of calibration and on screening and vaccination strategies used to illustrate the impact of parameter uncertainty, identified via calibration, on the uncertainty of policy-relevant outcomes. The appendix also provides details on other screening strategies implemented in the model useful in further policy analyses of cervical cancer prevention. Click here for file
