# Supplementary material for: Modeling human papillomavirus and cervical cancer in the United States for analyses of screening and vaccination
Source: Popul Health Metr. 2007 Oct 29;5:11. doi: 10.1186/1478-7954-5-11 (PMC2213637; doi:10.1186/1478-7954-5-11)
Supplement: Additional file 1 — An individual-based stochastic microsimulation of human papillomavirus and cervical cancer in the United States: Supplemental technical information. The appendix provided includes a supplementary description of the model structure, parameterization, calibration, and evaluation as well as information on the results of calibration and on screening and vaccination strategies used to illustrate the impact of parameter uncertainty, identified via calibration, on the uncertainty of policy-relevant outcomes. The appendix also provides details on other screening strategies implemented in the model useful in further policy analyses of cervical cancer prevention. [file 1478-7954-5-11-S1.doc]

**An individual-Based Stochastic Microsimulation of Human Papillomavirus and Cervical Cancer in the United States**

**Supplemental Technical Information**

**Model Structure, Parameterization, Calibration, Evaluation, Screening and Vaccination Strategies**

Accompanying the manuscript:

“Modeling human papillovirus and cerival cancer in the United States for analyses of screening and vaccination”

**Introduction**

In the following supplemental technical appendix, we describe a model of human papillomavirus (HPV) and cervical cancer. The appendix is structured as follows. First, we define terminology used in the description of our model in order to highlight its important attributes and to differentiate it from other modeling approaches that we and others have taken. Next, we describe the model’s structure as well as the details of its implementation and operation. We continue by describing the methods and data for the calibration of the model – defined here as the use of data from real-world studies to refine estimates of model inputs so that model outputs are more consistent with observed data than they were prior to calibration. We then describe the methods and data used in evaluating the external consistency and face validity of the model – defined here as the comparison of model predictions to data sources not used in calibration. Finally, we describe modules incorporated in the model that can simulate complex screening, diagnostic, and treatment algorithms as well as vaccination against high-risk, oncogenic HPV types.

**Modeling Taxonomies: Description of Model Attributes**

Because the meaning of terms used in taxonomies of model types often differ from study to study, we begin by clarifying the attributes of the model used for this analysis. Models can be categorized with respect to the following attributes: (1) whether key elements of a model, such as risks, change over time (dynamic) or not (static); (2) whether modeled events occur randomly (stochastic or probabilistic) or follow uniquely from specification of model parameter values (deterministic); (3) whether the population’s behavior in a model is simulated using aggregate variables for which values represent population averages (aggregate), or behaviors of each individual in the population are tracked separately (individual-based); (4) whether events are assumed to occur at discrete time intervals (discrete) or at any point on a continuum (continuous); (5) whether a model allows individuals to enter the model (open) or not (closed).

1. The distinction between static and dynamic models is typically used in reference to the treatment of key model parameters, such as the force of infection. In a static model, the force of infection is constant over calendar time (although may vary as a function of age or other individual-based factors), while in a dynamic model, the force of infection may change over time as a function of the prevalence of a disease of interest in the population. Although the model in this study may be linked directly to a dynamic transmission model, this link is not used for the present analysis. We allow for changes in the forces of type-specific infection as individuals age, to act as a proxy for the pattern of age at initiation of sexual activity and sexual behavior in U.S. women. We conduct sensitivity analyses for incidence parameters and we also rely on both our own dynamic transmission model and a review of the literature describing findings from other dynamic models, to inform our assumptions about the relative importance of herd immunity for policy questions in different situations.

2. A model is stochastic if events occur with allowance for “chance,” i.e., randomly. In contrast, in a deterministic model, all events occur in a pre-specified way that is fully determined by the parameter values and initial conditions of the model. A stochastic model can add computational complexity, but can allow for a more comprehensive evaluation of the impact of uncertainty. The model we describe here is stochastic. To avoid potential confusion, we distinguish between uncertainty and variability. The term “stochastic” is usually combined with the term “uncertainty” to indicate that in two or more situations (or simulations), different outcomes may occur simply by chance, even if the expectation of the outcome is the same. This uncertainty around possible outcomes, even under perfect knowledge of the rules or parameters that govern the expectations of these outcomes, is known as first-order uncertainty and is reflected in the model we use here. A second concept, known as second-order uncertainty, refers to uncertainty about parameter values themselves. Parameter values may be sampled from probability distributions reflecting imprecision or imperfect information about the values themselves. Our search across parameter space in the calibration of the model described below reflects an exploration of second-order uncertainty. In contrast to uncertainty, variability refers to the often “known” heterogeneity across subgroups or within a population (e.g., age or sex).

3. An aggregate model divides the population into a finite number of different compartments or states defined in terms of health status or other relevant variables, such as age groups. The numbers of people residing in the different model compartments or states change to reflect movements of individuals at the aggregate level (i.e., net changes that apply on average to the individuals residing in a particular compartment), and the model records the number of individuals in each compartment over time. One limitation of this approach is that the number of compartments quickly increases with complex diseases, as more variables are used to stratify the population in a model. For example, in our earlier models, reflecting different HPV types was less relevant to questions of screening frequency, and an aggregate model was quite suitable. Because we are assessing screening strategies that use HPV testing to detect high-risk types, and HPV-16/18 vaccination, stratification of states by HPV type is necessary for our current model. Consequently, many more health states would be needed to reflect this stratification in an aggregate model. Further, since prognosis, utilities, costs, and outcomes may differ based on individual factors, and since an individual’s history is likely an important determinant of her future probabilities, the number of required health states would continue to multiply. In contrast to aggregate models we have developed previously, the model described here is an individual-based model, often referred to as a microsimulation model. Since the unit of analysis is a single individual, the events and changes that occur for this individual are naturally simulated as stochastic processes (reflecting first-order uncertainty).

4. A model is continuous if changes occur continuously in time. In contrast, a discrete model only allows changes to occur at certain points in time when the model evaluates whether events occur or not. In this context, events include both transitions among health states (e.g., infection with HPV or death from cervical cancer) and potential public health or medical interventions (e.g., having a screening test performed or receiving treatment for CIN). The choice of continuous or discrete models may depend on considerations such as the process being modeled and the need to incorporate different types of information into the simulation. For discrete models, the interval between points in time when events can occur can either be constant or variable. Results from discrete models converge with those of continuous models as the size of the interval is made smaller. In this analysis, we use a discrete model with fixed time intervals of one month.

5. An open model allows individuals to enter and exit the model over calendar time, while a closed model does not allow for new individuals to enter. While dynamic transmission models are typically open, individual-based microsimulation models may or may not be. Our earlier models were closed deterministic models simulating a single birth cohort. Our dynamic transmission model allows individuals to enter the model via birth over time. The model used for this analysis can be utilized as either an open or a closed model. An open model, employing a population-based approach, can be very useful in assessing past trends and forecasting future trends in chronic noncommunicable diseases. Open models can be more data intensive (if one wants to account for parameters that change over time, e.g., secular trends) and can be more computationally intensive, depending on the complexity of the underlying structure. In this analysis, based on both data availability and the focus of comparison between strategies that have not yet been implemented to scale (e.g., vaccination), we use a closed model.

**Model Background and Overview**

We present the technical details of a model of HPV and cervical cancer in the United States. In addition to capturing the disease process, the model presented here also includes modules that simulate detailed screening and treatment strategies for the prevention of cervical cancer and its early detection. Another key module is used to simulate vaccination against specific, oncogenic HPV types. The model follows individual women throughout their lives, computing measures of health benefit including both life expectancy and quality-adjusted life expectancy. The model also records all costs associated with events such as vaccination, screening, diagnosis, treatment of precancer, and cancer care. By simulating a large number of individual women, expected health benefits and costs of alternative prevention policies that may include screening, vaccination or both can be assessed. The model calibration method used also allows the uncertainty in these assessments to be highlighted.

The model used for this analysis is a first-order Monte Carlo microsimulation. Specifically, it is a static, individual-based, stochastic model that can be run as an open or a closed model, and can be used in conjunction with a dynamic transmission model. In this analysis, the model is run as a *closed* model without direct linkage to the transmission model.

The model described here is one of a series of models we have developed which follow from earlier published deterministic Markov and semi-Markov cohort models [1-7]. For this analysis, we use a microsimulation model because it easily accommodates the tracking of individual behavior and allows individual attributes (e.g., age, vaccination status, screening compliance) and individual history (e.g., prior clinical events such as CIN) to affect future screening behavior, prognosis, and outcomes. Like some of our prior models, this one reflects multiple HPV types, both vaccine-targeted types and non-targeted types, and allows for exploration of the potential increase in the proportion of disease expected from non-targeted HPV types (i.e., type replacement). This latter feature contrasts to a dynamic transmission model developed by this team that can appropriately represent herd immunity effects, but only reflects vaccine-targeted HPV types 16 and 18. The model presented here is coded in the C++ programming language and can be run under both Microsoft Windows and UNIX operating systems.

# **Model Development Process**

The model development process included definition of model structure, parameterization, calibration, and evaluation of model performance. First, the model structure was designed to be consistent with current biological understanding of cervical carcinogenesis. Next, parameter ranges for model inputs for natural history were defined from longitudinal cohort studies. Given uncertainty in model inputs, observed epidemiologic data on outcomes such as age-specific HPV prevalence and cervical cancer incidence prior to widespread screening were used to define targets for calibration of these model parameters in the absence of screening. For calibration, input parameter values were sampled from the defined ranges, and simulations undertaken for each sampled parameter set. The goodness-of-fit (GOF) of modeled outputs resulting from each candidate set of input parameter values was evaluated using likelihood-based scores calculated based on the full array of calibration targets. We identified a subset of the sampled parameter combinations that had GOF scores that were statistically indistinguishable from that of the best-fitting set. Evaluation of the model performance was undertaken by comparing the modeled outcomes in the presence of screening to those in large, population-based studies.

**Model Structure**

The stochastic microsimulation of the natural history of HPV infection and cervical carcinogenesis simulates the transitions of individuals between a set of mutually-exclusive health states (**Appendix Figure 1**). In the model, HPV infection is stratified into 5 categories: not infected; HPV-16; HPV-18; other high-risk types (category includes types 31, 33, 35, 39, 45, 51, 52, 56, 58, 59, 66, 68, 73, 82); and low-risk types. Cervical Intraepithelial Neoplasia (CIN) status is modeled in three categories: no CIN; CIN1, and CIN2,3. Individual females enter the model at age 9 prior to sexual debut and remain in the model for the entirety of their lives. Transitions between health states occur at monthly intervals and depend on HPV type, age, history of prior HPV infection, type-specific natural immunity, previously-treated CIN, and screening patterns. Each month, a woman has an age- and type-specific probability of being infected with HPV. Modeled probabilities of age-related HPV infection act as a proxy for the probability of being sexually active combined with the probability of transmission and distribution of HPV types among sexual partners. The model can be enabled to consider indirect effects on health outcomes and costs associated with herd immunity by being linked to the output of a dynamic transmission model. Most women with HPV infections will develop transient abnormalities reflecting productive HPV infection, and some will progress to CIN2,3. Women infected with high-risk HPV types and persistent high-grade CIN may progress to invasive cancer, and those with invasive cancer can develop symptoms or progress to the next stage of cancer. We assume that symptomatic women with invasive cancer receive stage-specific treatment for their disease and are subject to the corresponding stage-specific survival rates. From every health state and in every month, women face competing mortality risks from all other causes.

**Appendix Figure 1.** Schematic of Natural History Model

***Model Schematic.*** *Each ellipse represents a state in the natural history model. HPV infection is stratified by type. Each month, a woman has a chance to transition from her current state to another state (transitions denoted by the dark black arrows) or else to remain in her current state. All women also have a chance of dying from all-cause mortality, and women with invasive cancer have an additional stage-specific chance of dying from their cancer.*

**Implementing the Model: A Static, Closed, First-Order, Monte Carlo Microsimulation**

As described above, a microsimulation model is one type of computer simulation model used to approximate a complex system or process. Such models can be designed both to replicate and to predict complex, real-world systems over time. The simulation model necessarily makes simplifying assumptions in its representation of how the real-world system operates, and describes the system using logical and/or mathematical relationships. Theory and data from multiple sources can be integrated in the design of a simulation model. The “simulation” is the generation of random paths through the model’s states over time. Relevant model output includes statistics about these paths such as the ordering of states in a path or time spent in a particular state. To design and specify a microsimulation, relevant characteristics that describe the system or process need to be identified, and the states and events controlling the transitions must be defined. In the case of disease simulation, aspects such as disease acquisition and progression must be specified. Relevant outputs for evaluating the system must also be identified. Experimentation by varying inputs and assumptions and observing the effects on the outputs can be performed.

In our model, during each monthly cycle, random numbers between 0 and 1 are used to determine the course of the currently-simulated woman’s disease progression, chance of dying, likelihood of screening/treatment, and success of interventions, as well other events that contain an element of uncertainty. The likelihood of a transition between health states is binomially distributed. If the random number generated falls between 0 and the probability of the event occurring, then the event occurs; if it falls between the probability and one, then the event does not occur. For example, if there is a 40% chance of an event occurring and the random number generated is 0.39, then the event occurrs; if the random number is 0.56, then the event does not occur. Thus, the closer a probability is to 1, the greater the chance the event does occur.

In this model we use the Mersenne Twister to generate pseudo-random numbers [8]. This generator is computationally efficient and generates a long “period”, or unique sequence of numbers. The simulation technique of "common random numbers" (CRN) is also employed in the model to reduce variation in model output induced by the random number generator across runs of the simulation model, and allowing the model to perform counterfactual experiments (i.e., the same women exposed to different interventions) [9,10]. This variance reduction feature allows the simulation of smaller cohorts of women (one million versus ten million women per cohort) without compromising accuracy. The model incorporates separate instantiations of the random number generator to allow for distinct, independent sequences, by controlling independent, person- and CIN-level events such as risk factors or CIN onset and progression. Given the same seeds for these random number sequences, identical person and CIN characteristics can be generated across runs. The variance across runs is then primarily induced by changes in input parameters, and the stochastic noise from the pseudorandom number streams is minimized. While simulation noise still exists, the noise is correlated across model runs, which is important in considering very small differences in effect (e.g., the incremental benefits of one screening strategy compared to another screening strategy). Thus the use of CRN reduces simulation noise without increasing sample size. Further, counterfactual experiments can be readily performed as each simulated person can act as his/her own “control”. For example, for a given screening strategy over-diagnosis can be computed by comparing at an individual level the time of detection in the presence of screening and the time of detection for the same neoplasia in the absence of screening.

As a Monte Carlo microsimulation, the process of running the model involves simulation of multiple individual women over the course of their lives, one by one until a population of a pre-specified size has been reached (**Appendix Figure 2**). Once all individual women have been simulated, estimates of various aggregate-level statistics are produced such as life expectancy, quality-adjusted life expectancy, cases of detected invasive cervical cancer, and costs.

**Appendix Figure 2.** Flowchart of Monte Carlo Simulation

***Flowchart of Monte Carlo Simulation.*** *The model simulation begins by loading data files containing transition probabilities, death rates, and other control parameters such as the required population size. Population statistics such as life expectancy and cumulative cancer risk are then initialized. Then, until the required population size is reached, the next woman enters the model. Each life of her month is simulated, including transitions to HPV infection and potential lesion growth as well as events such as screening, diagnosis, and treatment. On the month that the woman dies, data about her life are recorded. When the population has reached the required size, statistics are tallied and computed. Results are reported in output files, and the simulation ends.*

**Model Parameterization**

Parameterization of the model required specification of all age-specific (where appropriate) monthly transition probabilities comprising the natural history model summarized in **Appendix Figure 1**. Age-specific values for the transition probabilities are specified by combining a curve that determines the relative values as a function of age with a scalar multiplier that determines the level of all points on the curve. Uncertainty around parameter values is operationalized by searching across ranges for the scalar multipliers, while holding the age pattern constant. Age patterns were derived from published longitudinal studies and are described below in **Appendix Figures 3 through 11**. Ranges for scalar multipliers were defined to be broadly inclusive of study-reported confidence intervals, the highest and lowest estimate reported from different data sources, and expert opinion, and are summarized below in **Appendix Table 1**.

***Age Patterns for Model Transitions***

The following are a set of figures summarizing age functions for the model transitions. The figures describe the inputs in terms of transition rates (per 1,000 person-years or per 100,000 person-years as appropriate), which are translated into probabilities for the model simulations. Each transition rate corresponds to an arrow between two health states shown in the model schematic (**Appendix Figure 1**).

**Appendix Figure 3** shows the type/age-specific rates of HPV infection used in the model (i.e., the transitions in the model schematic from No HPV/No CIN to HPV Infected/No CIN) [7,11-28]. These transitions represent the force of infection and are independent between HPV types. In the model, type-specific immunity acts to reduce the corresponding type-specific infection rate. Because of this, the type-specific incidence of infection (a model output incorporating immunity and the force of infection) is not the same as the corresponding infection type-specific rate (a model input).

**Appendix Figure 3.**

**Appendix Figure 4** shows the type/age-specific rates of transition from HPV infection without CIN to CIN1 used in the model (i.e., the transitions in the model schematic from HPV Infected/No CIN to HPV Infected/CIN1) [7,18,22,29-31].

**Appendix Figure 4.**

**Appendix Figure 5** shows the type/age-specific rates of transition from HPV Infected or CIN1 to CIN2,3 used in the model (i.e., the transitions in the model schematic from HPV Infected/No CIN to HPV Infected/CIN2,3 and the transitions in the model schematic from HPV Infected/CIN1 to HPV Infected/CIN2,3) [7,32-40].

**Appendix Figure 5.**

**Appendix Figure 6** shows the type/age-specific rates of transition from CIN2,3 to Cerivcal Cancer used in the model (i.e., the transitions in the model schematic from HPV Infected/CIN2,3 to Local Cancer/Undetected) [41]. Infection with high-risk HPV is considered necessary for progression to invasive cancer.

**Appendix Figure 6.**

**Appendix Figure 7** shows the stage-specific rates of transition from less severe to more severe stages of Cerivcal Cancer used in the model (i.e., the transitions in the model schematic from Local Cancer/Undetected to Regional Cancer/Undetected and from Regional Cancer/Undetected to Distant Cancer/Undetected).

**Appendix Figure 7.**

**Appendix Figure 8** shows the age-specific rates of transition from CIN2,3, CIN1, and HPV Infected to Normal used in the model (i.e., the transitions in the model schematic from HPV Infected/CIN2,3 to No HPV/No CIN; from HPV Infected/CIN1 to No HPV/No CIN; and from HPV Infected/No CIN to No HPV/No CIN). Transitions from CIN to Normal were derived from multiple sources [7,18,22,31,38,42]. Transitions from HPV Infected to Normal were derived from additional sources as well [7,18,22,43]. It is assumed that of those individuals regressing from CIN2,3: 70% clear their HPV infection as well as their CIN, 15% clear their CIN but not their HPV infection, and 15% do not completely clear their CIN, instead regressing to CIN1 and retaining their HPV infection.

**Appendix Figure 8.**

**Appendix Figure 9** shows the stage-specific rates of becoming detected with cancer due to symptoms used in the model (i.e., the transitions in the model schematic from Local Cancer/Undetected to Local Cancer/Detected; from Regional Cancer/Undetected to Regional Cancer/Detected; and from Distant Cancer/Undetected to Distant Cancer/Detected) [7,41].

**Appendix Figure 9.**

**Appendix Figure 10** and **Appendix Figure 11** show age-specific all cause mortality rates and stage-specific mortality rates from cancer. From all health states and in every month, women are exposed to the appropriate age-specific all cause mortality rate. Additionally women with cervical cancer are also exposed to the appropriate stage-specific mortality rate. The mortality rates are derived from a variety of sources [7,41,44]. For women with cancer, the mortality rates are assumed to be additive with the percentage of women dying from cancer versus all other causes being equal to the cancer-specific mortality rate divided by the sum of the cancer-specific mortality rate and the all cause mortality rate.

**Appendix Figure 10.**

**Appendix Figure 11.**

***Level Parameters for Model Transitions: Scalar Multipliers***

Because of uncertainty about the estimated transition rates (model inputs) within each study and heterogeneity in the quantities estimated across studies due to methodological and population differences, wide plausible ranges were defined around the model input values depicted in the figures above. The upper and lower bound of each range were defined to be broadly inclusive of study-reported confidence intervals, the highest and lowest estimate reported from different data sources, and expert opinion. These ranges were operationalized as the product of the model input values depicted above and corresponding ranges of scalar multipliers (pre-calibration ranges). Calibration to a broad set of targets was performed to narrow the pre-calibration ranges of multipliers in a manner consistent with available observed, epidemiologic data.

**Appendix Table 1** describes the plausible ranges defined for the scalar multipliers used in the model. These plausible ranges around input parameter values comprise a joint uniform prior distribution for the inputs for purposes of calibration, as described below. While most input parameters were assumed to be independent from one another, we constrained our search for good-fitting parameter sets by requiring that the sets we generated have certain relationships. For example, for each HPV type, the regression rate from CIN1 to Normal was constrained to be equal to the regression rate from CIN1 to HPV Infected.

**Appendix Table 1**. Input Search Ranges for Parameter Multipliers (Prior Distributions)

| **Progression Parameters [Type-specific Stratum]** | **Search Range** | **Searching Constraint** |
| --- | --- | --- |
| Normal 🡪 HPV Infected [Low-Risk HPV] | 1.0 – 4.0 |  |
| Normal 🡪 HPV Infected [Other High-Risk HPV] | 1.0 – 8.0 |  |
| Normal 🡪 HPV Infected [HPV-16] | 1.0 – 8.0 | > Normal 🡪 HPV Infected [HPV-18] |
| Normal 🡪 HPV Infected [HPV-18] | 1.0 – 8.0 | > Normal 🡪 HPV Infected [Other High-Risk HPV] |
| HPV 🡪 CIN1 [Low-Risk HPV] | 0.1 – 6.0 |  |
| HPV 🡪 CIN1 [Other High-Risk HPV] | 0.1 – 6.0 |  |
| HPV 🡪 CIN1 [HPV-16] | 0.1 – 6.0 |  |
| HPV 🡪 CIN1 [HPV-18] | 0.1 – 6.0 |  |
| HPV 🡪 CIN2,3 [Low-Risk HPV] | 0.0 – 0.1 |  |
| HPV 🡪 CIN2,3 [Other High-Risk HPV] | 0.0 – 0.1 |  |
| HPV 🡪 CIN2,3 [HPV-16] | 0.1 – 1.0 |  |
| HPV 🡪 CIN2,3 [HPV-18] | 0.0 – 0.1 |  |
| CIN1 🡪 CIN2,3 [Low-Risk HPV] | 0.5 – 4.0 |  |
| CIN1 🡪 CIN2,3 [Other High-Risk HPV] | 0.1 – 4.0 |  |
| CIN1 🡪 CIN2,3 [HPV-16] | 0.5 – 6.0 |  |
| CIN1 🡪 CIN2,3 [HPV-18] | 0.1 – 4.0 |  |
| CIN2,3 🡪 cancer [Other High-Risk HPV] | 1.0 – 3.0 |  |
| CIN2,3 🡪 cancer [HPV-16] | 1.0 – 5.0 |  |
| CIN2,3 🡪 cancer [HPV-18] | 1.0 – 5.0 |  |

| **Regression Parameters [Type-specific Stratum]** | **Search Range** | **Searching Constraint** |
| --- | --- | --- |
| HPV 🡪 Normal [Low-Risk HPV] | 1.5 – 6.0 |  |
| HPV 🡪 Normal [Other High-Risk HPV] | 1.5 – 6.0 |  |
| HPV 🡪 Normal [HPV-16] | 1.5 – 6.0 |  |
| HPV 🡪 Normal [HPV-18] | 1.5 – 6.0 | = HPV Infected 🡪 Normal [HPV-16] |
| CIN1 🡪 Normal [Low-Risk HPV] | 0.5 – 5.0 |  |
| CIN1 🡪 Normal [Other High-Risk HPV] | 0.5 – 5.0 |  |
| CIN1 🡪 Normal [HPV-16] | 0.5 – 5.0 |  |
| CIN1 🡪 Normal [HPV-18] | 0.5 – 5.0 | = CIN1 🡪 Normal [HPV-16] |
| CIN1 🡪 HPV [Low-Risk HPV] | 0.5 – 5.0 | = CIN1 🡪 Normal [Low-Risk HPV] |
| CIN1 🡪 HPV [Other High-Risk HPV] | 0.5 – 5.0 | = CIN1 🡪 Normal [Other High-Risk HPV] |
| CIN1 🡪 HPV [HPV-16] | 0.5 – 5.0 | = CIN1 🡪 Normal [HPV-16] |
| CIN1 🡪 HPV [HPV-18] | 0.5 – 5.0 | = CIN1 🡪 Normal [HPV-18] |
| CIN2,3 🡪 Normal [Low-Risk HPV] | 0.5 – 5.0 |  |
| CIN2,3 🡪 Normal [Other High-Risk HPV] | 0.5 – 5.0 |  |
| CIN2,3 🡪 Normal [HPV-16] | 0.5 – 5.0 |  |
| CIN2,3 🡪 Normal [HPV-18] | 0.5 – 5.0 |  |
| CIN2,3 🡪 HPV [Low-Risk HPV] | 0.5 – 5.0 | = CIN2,3 🡪 Normal [Low-Risk HPV] |
| CIN2,3 🡪 HPV [Other High-Risk HPV] | 0.5 – 5.0 | = CIN2,3 🡪 Normal [Other High-Risk HPV] |
| CIN2,3 🡪 HPV [HPV-16] | 0.5 – 5.0 | = CIN2,3 🡪 Normal [HPV-16] |
| CIN2,3 🡪 HPV [HPV-18] | 0.5 – 5.0 | = CIN2,3 🡪 Normal [HPV-18] |
| CIN2,3 🡪 CIN1 [Low-Risk HPV] | 0.5 – 5.0 | = CIN2,3 🡪 Normal [Low-Risk HPV] |
| CIN2,3 🡪 CIN1 [Other High-Risk HPV] | 0.5 – 5.0 | = CIN2,3 🡪 Normal [Other High-Risk HPV] |
| CIN2,3 🡪 CIN1 [HPV-16] | 0.5 – 5.0 | = CIN2,3 🡪 Normal [HPV-16] |
| CIN2,3 🡪 CIN1 [HPV-18] | 0.5 – 5.0 | = CIN2,3 🡪 Normal [HPV-18] |

| **Immunity [Type-specific Stratum]** | **Search Range** | **Searching Constraint** |
| --- | --- | --- |
| Immune Degree [Other High-Risk HPV] | 0.0 – 1.0 |  |
| Immune Degree [HPV-16] | 0.0 – 1.0 | > Immune Degree [Other High-Risk HPV] |
| Immune Degree [HPV-18] | 0.0 – 1.0 | > Immune Degree [Other High-Risk HPV] |

**Model Calibration**

We define calibration of the model as the use of data from real-world studies to refine estimates of model inputs so that model outputs are more consistent with observed data than they were prior to calibration. Our approach to model calibration was based on a multi-dimensional random search in which input parameter sets (i.e., sets of values each consisting of one multiplier for each model input being searched) were sampled from plausible ranges defined as described above, and then separate simulations of the model were run using each set of the sampled parameter values. Outputs from the model were compared to epidemiologic data, with the likelihood of the input parameter set generating the modeled output increasing as the correspondence between modeled outputs and epidemiologic data became more precise. A flowchart of model calibration is shown in **Appendix Figure 12**.

**Appendix Figure 12.** Flowchart of Multi-dimensional Search Used in Model Calibration

***Calibration Schematic.*** *Files that control the boundaries on the mult-parameter random search as well as other input files are loaded. A parameter set is randomly generated and used to simulate a cohort of women (see* ***Appendix Figures 1 and 2*** *for details on the simulation of each cohort). The outputs from this simulation are recorded. This process is repeated until the required number of parameter sets has been simulated. Parameter sets and their associated calibration outputs are output to a file for scoring with respect to real-world targets derived from studies.*

***Model Calibration Targets***

We defined 84 epidemiologic outcomes that comprised targets for calibration, within the following categories: type- and age-specific prevalence of HPV; type- and age-specific duration of HPV infections; age-specific prevalence of CIN1 and CIN2,3; age-specific cancer incidence; lifetime cancer risk; and HPV type-distribution within CIN and cancer. For each calibration target, we determined a 95% confidence interval using data arising from population-based studies. When multiple data sources were available, we used random-effects models for data synthesis to produce combined point estimates and confidence intervals [45]. For some calibration targets, for which the quantities of interest were expected to vary across epidemiologic settings, we limited the range of data sources that informed the definition of our targets. For example, targets on HPV and CIN prevalence were based only on North American studies, despite availability of a wider range of possible data sources. Because the model is intended for policy analyses of screening and vaccination in the United States, targets relating to cancer were based exclusively on data from the U.S.

To establish calibration targets for *age-specific prevalence of HPV and CIN* we included studies that provided sufficient information on sample size and prevalence of age-specific infection with high-risk or low-risk HPV types [14,46-61]. Similarly, we included only studies that provided sufficient information on sample size and prevalence of CIN1 or CIN2,3 [14,46,48,62-71]. For targets relating to *duration of infection*, data were derived from a single, longitudinal Brazilian study that collected frequent, repeated measures of HPV status in a large cohort of women over an average follow-up of 53 months [42,43], supplemented with secondary data from studies with shorter periods of observation from the U.S. To evaluate model outcomes on the natural history of disease in the absence of screening, targets on the *age-specific incidence of invasive cervical cancer* were defined based on 1959-60 data reported from multiple U.S. registries to the International Agency for Research on Cancer (IARC) [72]. Observations from each registry were treated as outcomes of independent experiments and combined using a random-effects model [73]. To define targets on the *lifetime risk of cancer*, we incorporated the lower and upper confidence intervals from the age-specific cancer incidence rates and all-cause, age-specific U.S. mortality rates in a multiple-decrement life table approach to account for competing mortality hazards [44,74,75]. We defined targets on *HPV type distribution in CIN and cancer* based primarily on systematic reviews, supplemented by subsequently-published studies [23,58,76-97]. Specifically, we estimated the proportion of patients with CIN2,3 infected with HPV-16, HPV-18, or another high-risk HPV type; the proportion of patients with CIN1 infected with HPV-16/18 or another high-risk type; and the proportion of patients with invasive cervical cancer infected with either HPV-16 or HPV-18.

***Calibration Implementation***

As described above, parameter values governing each transition probability were specified based on an age pattern combined with a scalar multiplier determining the level of all points on the age curve. Uncertainty around transitions was operationalized by specifying distributions around the scalar multipliers (summarized in **Appendix Table 1**), collectively comprising a joint prior distribution for the model parameters.

We sampled randomly from this joint prior distribution (i.e., generating input parameter sets) and simulated a population of 100,000 individuals in the model for each set of sampled parameter values. A total of 1,000,000 parameter sets were sampled and simulated. For each set of sampled parameter values, we compared the modeled outputs corresponding to each of the 84 calibration targets to the data-derived target. The formal comparison was based on a likelihood score, computed under the assumption that calibration targets were characterized by independent, normal probability density functions, with means and standard deviations derived from the empirical 95% confidence intervals. An overall goodness-of-fit (GOF) score was computed as negative two times the sum of the log-likelihood scores for each target. To compare the fit of different parameter sets, we assumed that the distribution of GOF scores may be approximated by a chi-square distribution with the number of degrees of freedom equal to the number of calibration targets.

With likelihood-based GOF scores, we identified multiple parameter set combinations whose outputs were simultaneously consistent with calibration targets derived from epidemiologic data. First, we determined our best-fitting parameter set as the one with the lowest GOF score – the model-generated input parameter set whose simulated outputs were simultaneously closest to all calibration targets. We identified those parameter sets with GOF scores that were statistically indistinguishable from the GOF score of the best-fitting set (based on a likelihood ratio test with p<0.05), and considered these good-fitting. In addition to this first criterion of overall goodness-of-fit, we also evaluated candidate parameter sets based on a second criterion, goodness-of-fit with respect to a subset of high-priority targets, based on their relevance to policy questions regarding vaccination and screening. To provided the best fit to the high-priority targets, we computed a GOF subscore based on the following high-priority targets: prevalence of CIN1 at ages 25-29 and 35-39 years, proportion of HPV-16/18 in CIN1, proportion of HPV-16 in CIN2,3 and cancer, proportion of HPV-18 in cancer, cancer incidence at ages 45-49, 55-59, and 70-74 years. Finally, we accepted those parameter sets that were good-fitting and had high-priority GOF subscores among the top 1% of all parameter sets. For efficiency, subsequent analyses were based on a random resample of 50 parameter sets from the array of accepted parameter sets to preserve the representation of overall parameter uncertainty while minimizing the computational intensity required for the simulations.

**Results of Model Calibration**

**Appendix Table 2** shows the results of the calibration to multiple epidemiologic targets on the ranges of scalar multipliers applied to the model input values. Pre-Calibration multipliers have mean values (over the 1,000,000 parameter sets searched) that are the mid-points of the ranges searched, reflecting their uniform prior distribution. Exceptions to this are those scalar multipliers constrained to be higher than other multipliers being searched (**Appendix Table 1**). For the subgroup of input parameter sets that were judged to be good-fitting with respect to epidemiological data, we recalculated the mean, standard deviation, and ranges of each multliplier. Then, we compared the change in the mean value of each scalar multiplier to the size of the range searched. Larger shifts in the mean values for the post-calibration, good-fitting multipliers represent the necessity of a greater change from the Pre-Calibration model input values in order to fit epidemiological data with modeled outputs. Multiplier values changed most for Low-Risk HPV infection; progression from Low-Risk HPV Infection to CIN1; progression from Other High-Risk HPV CIN1 to CIN2,3; progression from HPV-16 CIN2,3 to Cancer; regression from CIN1 for Other High-Risk HPV, HPV-16, and HPV-18; and regression from Low-Risk HPV Infection to Normal.

**Appendix Table 2.** Influence of Calibration on Scalar Multipliers of Model Inputs

|  | **Pre-Calibration** | | | | **Post-Calibration** | | | | **Difference in Pre-Calibration and Post-Calibration Means** |
| --- | --- | --- | --- | --- | --- | --- | --- | --- | --- |
|  |  |  |  |  |  |  |  |  | **Compared to Size of the Search Range** |
|  | **Mean** | **Std Dev.** | **Min** | **Max** | **Mean** | **Std. Dev.** | **Min** | **Max** | **%** |
| Type-specific Immunity |  |  |  |  |  |  |  |  |  |
| Other High-Risk | 0.500 | 0.288 | 0.00 | 1.00 | 0.543 | 0.206 | 0.00 | 0.88 | 4.3659 |
| HPV-16 | 0.750 | 0.221 | 0.00 | 1.00 | 0.785 | 0.138 | 0.21 | 1.00 | 3.5925 |
| HPV-18 | 0.749 | 0.221 | 0.00 | 1.00 | 0.786 | 0.168 | 0.07 | 1.00 | 3.6101 |
|  |  |  |  |  |  |  |  |  |  |
| HPV Infection |  |  |  |  |  |  |  |  |  |
| Low-Risk HPV | 2.501 | 0.866 | 1.00 | 4.00 | 3.234 | 0.578 | 1.19 | 4.00 | 24.4590 |
| Other High-Risk HPV | 4.506 | 2.019 | 1.00 | 8.00 | 4.901 | 1.801 | 1.17 | 8.00 | 5.6482 |
| HPV-16 | 7.128 | 1.022 | 1.20 | 8.00 | 7.285 | 0.780 | 3.21 | 8.00 | 2.3068 |
| HPV-18 | 6.256 | 1.539 | 1.01 | 8.00 | 6.552 | 1.250 | 2.74 | 8.00 | 4.2314 |
|  |  |  |  |  |  |  |  |  |  |
| HPV to CIN1 |  |  |  |  |  |  |  |  |  |
| Low-Risk | 3.050 | 1.704 | 0.10 | 6.00 | 3.828 | 1.404 | 0.23 | 6.00 | 13.1787 |
| Other High-Risk HPV | 3.053 | 1.701 | 0.10 | 6.00 | 2.778 | 1.436 | 0.21 | 5.99 | -4.6663 |
| HPV-16 | 3.044 | 1.702 | 0.10 | 6.00 | 3.128 | 1.610 | 0.13 | 5.98 | 1.4222 |
| HPV-18 | 3.054 | 1.703 | 0.10 | 6.00 | 2.777 | 1.567 | 0.12 | 5.98 | -4.6848 |
|  |  |  |  |  |  |  |  |  |  |
| CIN1 to CIN2,3 |  |  |  |  |  |  |  |  |  |
| Low-Risk HPV | 2.250 | 1.011 | 0.50 | 4.00 | 2.098 | 0.974 | 0.51 | 4.00 | -4.3320 |
| Other High-Risk HPV | 2.050 | 1.126 | 0.10 | 4.00 | 0.801 | 0.551 | 0.10 | 3.06 | -32.0141 |
| HPV-16 HPV | 3.253 | 1.588 | 0.50 | 6.00 | 3.392 | 1.508 | 0.51 | 5.99 | 2.5360 |
| HPV-18 HPV | 2.051 | 1.126 | 0.10 | 4.00 | 1.864 | 0.991 | 0.16 | 3.99 | -4.7939 |
|  |  |  |  |  |  |  |  |  |  |
| CIN2,3 to Cancer |  |  |  |  |  |  |  |  |  |
| Other High-Risk HPV | 2.000 | 0.577 | 1.00 | 3.00 | 1.966 | 0.579 | 1.00 | 3.00 | -1.6866 |
| HPV-16 | 3.000 | 1.155 | 1.00 | 5.00 | 3.451 | 0.962 | 1.23 | 4.99 | 11.2756 |
| HPV-18 | 3.002 | 1.155 | 1.00 | 5.00 | 3.356 | 1.017 | 1.04 | 5.00 | 8.8424 |
|  |  |  |  |  |  |  |  |  |  |
| CIN2,3 to CIN1 |  |  |  |  |  |  |  |  |  |
| Low-Risk HPV | 2.748 | 1.299 | 0.50 | 5.00 | 2.928 | 1.217 | 0.50 | 5.00 | 4.0050 |
| Other High-Risk HPV | 2.753 | 1.300 | 0.50 | 5.00 | 2.982 | 1.250 | 0.50 | 5.00 | 5.0782 |
| HPV-16 | 2.753 | 1.297 | 0.50 | 5.00 | 2.600 | 1.207 | 0.52 | 4.99 | -3.4139 |
| HPV-18 | 2.753 | 1.299 | 0.50 | 5.00 | 2.802 | 1.257 | 0.53 | 4.99 | 1.0924 |
|  |  |  |  |  |  |  |  |  |  |
| CIN2,3 to HPV |  |  |  |  |  |  |  |  |  |
| Low-Risk HPV | 2.748 | 1.299 | 0.50 | 5.00 | 2.928 | 1.217 | 0.50 | 5.00 | 4.0050 |
| Other High-Risk HPV | 2.753 | 1.300 | 0.50 | 5.00 | 2.982 | 1.250 | 0.50 | 5.00 | 5.0782 |
| HPV1-6 | 2.753 | 1.297 | 0.50 | 5.00 | 2.600 | 1.207 | 0.52 | 4.99 | -3.4139 |
| HPV-18 | 2.753 | 1.299 | 0.50 | 5.00 | 2.802 | 1.257 | 0.53 | 4.99 | 1.0924 |
|  |  |  |  |  |  |  |  |  |  |
| CIN2,3 to Normal |  |  |  |  |  |  |  |  |  |
| Low-Risk HPV | 2.748 | 1.299 | 0.50 | 5.00 | 2.928 | 1.217 | 0.50 | 5.00 | 4.0050 |
| Other High-Risk HPV | 2.753 | 1.300 | 0.50 | 5.00 | 2.982 | 1.250 | 0.50 | 5.00 | 5.0782 |
| HPV-16 | 2.753 | 1.297 | 0.50 | 5.00 | 2.600 | 1.207 | 0.52 | 4.99 | -3.4139 |
| HPV-18 | 2.753 | 1.299 | 0.50 | 5.00 | 2.802 | 1.257 | 0.53 | 4.99 | 1.0924 |
|  |  |  |  |  |  |  |  |  |  |
| CIN1 to HPV |  |  |  |  |  |  |  |  |  |
| Low-Risk HPV | 2.751 | 1.300 | 0.50 | 5.00 | 2.501 | 1.182 | 0.50 | 4.99 | -5.5377 |
| Other High-Risk HPV | 2.752 | 1.300 | 0.50 | 5.00 | 3.249 | 1.167 | 0.51 | 5.00 | 11.0488 |
| HPV-16 | 2.753 | 1.298 | 0.50 | 5.00 | 3.426 | 1.047 | 0.66 | 5.00 | 14.9626 |
| HPV-18 | 2.753 | 1.298 | 0.50 | 5.00 | 3.426 | 1.047 | 0.66 | 5.00 | 14.9626 |
|  |  |  |  |  |  |  |  |  |  |
| CIN1 to Normal |  |  |  |  |  |  |  |  |  |
| Low-Risk HPV | 2.751 | 1.300 | 0.50 | 5.00 | 2.501 | 1.182 | 0.50 | 4.99 | -5.5377 |
| Other High-Risk HPV | 2.752 | 1.300 | 0.50 | 5.00 | 3.249 | 1.167 | 0.51 | 5.00 | 11.0488 |
| HPV-16 | 2.753 | 1.298 | 0.50 | 5.00 | 3.426 | 1.047 | 0.66 | 5.00 | 14.9626 |
| HPV-18 | 2.753 | 1.298 | 0.50 | 5.00 | 3.426 | 1.047 | 0.66 | 5.00 | 14.9626 |
|  |  |  |  |  |  |  |  |  |  |
| HPV to Normal |  |  |  |  |  |  |  |  |  |
| Low-Risk HPV | 3.750 | 1.301 | 1.50 | 6.00 | 4.600 | 0.828 | 2.49 | 6.00 | 18.8870 |
| Other High-Risk HPV | 3.751 | 1.299 | 1.50 | 6.00 | 3.963 | 1.087 | 1.65 | 5.99 | 4.6932 |
| HPV-16 | 3.748 | 1.301 | 1.50 | 6.00 | 3.641 | 1.163 | 1.51 | 6.00 | -2.3709 |
| HPV-18 | 3.748 | 1.301 | 1.50 | 6.00 | 3.641 | 1.163 | 1.51 | 6.00 | -2.3709 |

While **Appendix Table 2** gives a sense of how the distributions of scalar multipliers shift due to calibration to epidemiological data, the joint distribution of multipliers also describes a correlation structure between different multipliers. First, **Appendix Table 3** shows 7 examples of the 50 good-fitting model input parameter sets used for screening and vaccination analyses.

**Appendix Table 3.** Examples of Good-Fitting Calibrated Parameter Sets Used in Screening and Vaccination Analyses

|  | **Example Parameter**  **Set**  **1** | **Example Parameter**  **Set**  **2** | **Example Parameter**  **Set**  **3** | **Example Parameter**  **Set**  **4** | **Example Parameter**  **Set**  **5** | **Example Parameter**  **Set**  **6** | **Example Parameter**  **Set**  **7** |
| --- | --- | --- | --- | --- | --- | --- | --- |
| **Immunity [Other High-Risk HPV]** | 0.76255 | 0.49087 | 0.68173 | 0.13865 | 0.68533 | 0.57570 | 0.35831 |
| **Immunity [HPV-16]** | 0.78159 | 0.84336 | 0.79416 | 0.88348 | 0.71033 | 0.91107 | 0.64973 |
| **Immunity [HPV-18]** | 0.79260 | 0.83515 | 0.73617 | 0.25606 | 0.96692 | 0.84347 | 0.55803 |
| **Normal to HPV Infection [Low-Risk HPV]** | 3.75942 | 3.52746 | 3.44453 | 3.26397 | 3.77937 | 3.85959 | 3.84007 |
| **Normal to HPV Infection [Other High-Risk HPV]** | 7.69360 | 3.16249 | 3.60921 | 3.23017 | 7.74745 | 1.47932 | 2.85936 |
| **Normal to HPV Infection [HPV-16]** | 7.90374 | 7.52942 | 5.67974 | 5.96839 | 7.98387 | 7.97772 | 7.38222 |
| **Normal to HPV Infection [HPV-18]** | 7.86304 | 4.13439 | 4.45938 | 5.63714 | 7.96577 | 7.67153 | 6.84799 |
| **HPV Infection to CIN1 [Low-Risk HPV]** | 5.64263 | 2.37695 | 1.44953 | 4.01164 | 4.81061 | 2.9648 | 5.71892 |
| **HPV Infection to CIN1 [Other High-Risk HPV]** | 0.53494 | 2.48536 | 0.97772 | 5.49310 | 3.99002 | 3.14100 | 3.91345 |
| **HPV Infection to CIN1 [HPV-16]** | 3.96580 | 5.91631 | 0.27450 | 4.60823 | 1.54123 | 0.22951 | 2.86141 |
| **HPV Infection to CIN1 [HPV-18]** | 5.22357 | 1.52241 | 0.25862 | 1.06907 | 4.65208 | 2.63318 | 0.48881 |
| **HPV Infection to CIN2,3 [Low-Risk HPV]** | 0.01215 | 0.09307 | 0.01908 | 0.06004 | 0.01665 | 0.07070 | 0.03624 |
| **HPV Infection to CIN2,3 [Other High-Risk HPV]** | 0.04532 | 0.01368 | 0.00728 | 0.07351 | 0.00792 | 0.01400 | 0.04856 |
| **HPV Infection to CIN2,3 [HPV-16]** | 0.70912 | 0.59743 | 0.85537 | 0.47598 | 0.41403 | 0.86974 | 0.46634 |
| **HPV Infection to CIN2,3 [HPV-18]** | 0.03472 | 0.05472 | 0.02946 | 0.03258 | 0.05018 | 0.05601 | 0.06406 |
| **CIN1 to CIN2,3 [Low-Risk HPV]** | 2.70561 | 1.85552 | 0.61789 | 3.95525 | 1.46490 | 1.67090 | 2.72910 |
| **CIN1 to CIN2,3 [Other High-Risk HPV]** | 0.92510 | 0.87130 | 0.80501 | 0.34869 | 0.79467 | 1.81175 | 0.27284 |
| **CIN1 to CIN2,3 [HPV-16]** | 1.80363 | 3.74183 | 1.25168 | 5.32351 | 3.86039 | 1.05974 | 1.16760 |
| **CIN1 to CIN2,3 [HPV-18]** | 2.48921 | 3.58559 | 1.27776 | 2.13360 | 2.65035 | 0.67290 | 3.09165 |
| **CIN2,3 to Cancer [Other High-Risk HPV]** | 1.36719 | 1.44676 | 1.51769 | 2.10634 | 2.48943 | 2.11831 | 1.55290 |
| **CIN2,3 to Cancer [HPV-16]** | 4.58147 | 2.43661 | 2.58937 | 3.97031 | 2.99669 | 2.52023 | 4.95265 |
| **CIN2,3 to Cancer [HPV-18]** | 1.99302 | 4.75057 | 4.50561 | 2.71858 | 2.46722 | 4.84922 | 1.83259 |
| **CIN2,3 to Normal [Low-Risk HPV]** | 3.78646 | 4.16897 | 3.86793 | 0.75417 | 2.13744 | 3.86180 | 2.18349 |
| **CIN2,3 to Normal [Other High-Risk HPV]** | 1.77839 | 4.92900 | 3.24280 | 3.16154 | 2.92421 | 1.76770 | 0.55371 |
| **CIN2,3 to Normal [HPV-16]** | 0.70195 | 2.10859 | 4.50181 | 3.78431 | 3.31652 | 2.45198 | 1.61720 |
| **CIN2,3 to Normal [HPV-18]** | 4.95033 | 2.68882 | 3.54804 | 4.09323 | 4.93019 | 4.55211 | 1.76025 |
| **CIN1 to Normal [Low-Risk HPV]** | 2.36324 | 0.93287 | 0.71699 | 4.37914 | 1.94689 | 1.33516 | 3.42531 |
| **CIN1 to Normal [Other High-Risk HPV]** | 1.37026 | 3.63699 | 0.83686 | 3.83007 | 4.02886 | 2.67997 | 2.68928 |
| **CIN1 to Normal [HPV-16]** | 4.63685 | 3.03735 | 0.66253 | 3.51115 | 4.07349 | 2.10568 | 1.97239 |
| **CIN1 to Normal [HPV-18]** | 4.63685 | 3.03735 | 0.66253 | 3.51115 | 4.07349 | 2.10568 | 1.97239 |
| **HPV Infection to Normal [Low-Risk HPV]** | 5.93843 | 4.30358 | 3.95128 | 4.51887 | 3.73629 | 4.00802 | 3.03030 |
| **HPV Infection to Normal [Other High-Risk HPV]** | 3.71069 | 3.20533 | 3.27699 | 5.84408 | 4.88586 | 5.22587 | 5.75864 |
| **HPV Infection to Normal [HPV-16]** | 4.03005 | 5.61214 | 1.79052 | 2.25401 | 2.33418 | 2.19040 | 4.36089 |
| **HPV Infection to Normal [HPV-18]** | 4.03005 | 5.61214 | 1.79052 | 2.25401 | 2.33418 | 2.19040 | 4.36089 |

In the Pre-Calibration parameter, no correlations exist between parameter values except those introduced by search constraints (see **Appendix Table 1**). However, in the Post-Calibration parameter sets, the joint distribution of good-fitting multiplier values provides insight into correlations between model values necessary to fit model outputs to epidemiologic data. For example, it may be the case that when progression from HPV Infection to CIN1 is high for a given type, regression from CIN1 to Normal may also need to be high for that HPV type in order to fit epidemiologic data. Another example might be that when progression from HPV to CIN1 for one HPV type is high, the corresponding progression from HPV to CIN1 for another HPV type must also be high. **Appendix Tables 4A, B, and C** show the pairwise correlation coefficients for the model input parameters in good-fitting parameter sets. Coefficients are tested for significance at the p<0.05 level using a Bonferroni correction. Significant correlation coefficients are starred and shown with a shaded background. General themes that can be seen in the tables include: 1) Immunity levels across types are positively correlated; 2) Risk of infection is positively correlated across types and also with immunity level; 3) Immunity is positively correlated with rates of progression from HPV to CIN1 and CIN23; 4) Immunity is negatively correlated with clearance of HPV; 5) Progression to CIN1 is positively correlated with regression from CIN1 within HPV type.

**Appendix Table 4A.** Pairwise Correlation of Calibrated Model Inputs

|  | **Immunity [Other High-Risk HPV]** | **Immunity [HPV-16]** | **Immunity [HPV-18]** | **Normal to HPV Infection [Low-Risk HPV]** | **Normal to HPV Infection [Other High-Risk HPV]** | **Normal to HPV Infection [HPV-16]** | **Normal to HPV Infection [HPV-18]** | **HPV Infection to CIN1 [Low-Risk HPV]** | **HPV Infection to CIN1 [Other High-Risk HPV]** | **HPV Infection to CIN1 [HPV-16]** | **HPV Infection to CIN1 [HPV-18]** |
| --- | --- | --- | --- | --- | --- | --- | --- | --- | --- | --- | --- |
| **Immunity [Other High-Risk HPV]** | 1 |  |  |  |  |  |  |  |  |  |  |
| **Immunity [HPV-16]** | 0.4379* | 1 |  |  |  |  |  |  |  |  |  |
| **Immunity [HPV-18]** | 0.5026* | 0.2041* | 1 |  |  |  |  |  |  |  |  |
| **Normal to HPV Infection [Low-Risk HPV]** | 0.0086 | -0.0063 | -0.0235 | 1 |  |  |  |  |  |  |  |
| **Normal to HPV Infection [Other High-Risk HPV]** | 0.5265* | 0.2636* | 0.3246* | -0.0686 | 1 |  |  |  |  |  |  |
| **Normal to HPV Infection [HPV-16]** | 0.3038* | 0.1719 | 0.2445* | -0.116 | 0.4508* | 1 |  |  |  |  |  |
| **Normal to HPV Infection [HPV-18]** | 0.3893* | 0.2000* | 0.2986* | -0.1088 | 0.6069* | 0.7345* | 1 |  |  |  |  |
| **HPV Infection to CIN1 [Low-Risk HPV]** | -0.0814 | -0.0987 | -0.0646 | -0.1048 | -0.1367 | -0.0448 | -0.0481 | 1 |  |  |  |
| **HPV Infection to CIN1 [Other High-Risk HPV]** | 0.1339 | 0.1216 | 0.0428 | -0.0973 | -0.0931 | -0.0146 | 0.0047 | -0.0151 | 1 |  |  |
| **HPV Infection to CIN1 [HPV-16]** | -0.0051 | 0.2375* | 0.0136 | -0.0571 | 0.1138 | -0.0122 | 0.0761 | -0.0069 | 0.1011 | 1 |  |
| **HPV Infection to CIN1 [HPV-18]** | 0.0042 | -0.1122 | 0.12 | 0.0692 | 0.0703 | 0.0247 | -0.0488 | 0.0327 | -0.0155 | -0.0506 | 1 |
| **HPV Infection to CIN2,3 [Low-Risk HPV]** | -0.0085 | 0.0427 | -0.0105 | -0.0286 | -0.0719 | -0.0666 | -0.0769 | -0.0097 | -0.0218 | 0.02 | 0.0773 |
| **HPV Infection to CIN2,3 [Other High-Risk HPV]** | -0.0013 | -0.0227 | -0.0149 | -0.0222 | -0.023 | -0.0024 | -0.0042 | 0.1126 | 0.104 | -0.0453 | 0.0032 |
| **HPV Infection to CIN2,3 [HPV-16]** | 0.0954 | 0.1436 | 0.0243 | 0.0154 | 0.0946 | 0.0281 | 0.0454 | 0.018 | 0.0111 | -0.003 | 0.1402 |
| **HPV Infection to CIN2,3 [HPV-18]** | 0.0043 | 0.021 | 0.0293 | -0.0214 | 0.0528 | -0.0153 | 0.0105 | 0.0427 | 0.0453 | -0.0518 | -0.0964 |
| **CIN1 to CIN2,3 [Low-Risk HPV]** | -0.0142 | 0.005 | -0.0033 | -0.0573 | 0.0521 | 0.0919 | 0.0992 | 0.0153 | 0.025 | 0.0333 | -0.051 |
| **CIN1 to CIN2,3 [Other High-Risk HPV]** | 0.1919* | 0.0482 | 0.1434 | 0.073 | -0.0665 | -0.0288 | -0.0257 | 0.0368 | -0.1723 | -0.1052 | -0.0658 |
| **CIN1 to CIN2,3 [HPV-16]** | 0.0801 | 0.2303* | 0.0719 | 0.0352 | 0.0844 | 0.0521 | 0.0477 | -0.053 | -0.0959 | 0.0135 | 0.1453 |
| **CIN1 to CIN2,3 [HPV-18]** | -0.0991 | -0.0633 | 0.0667 | -0.0184 | -0.0388 | -0.0429 | -0.0482 | 0.0331 | -0.1509 | 0.2371* | -0.4133* |
| **CIN2,3 to Cancer [Other High-Risk HPV]** | -0.1141 | -0.1432 | -0.0692 | 0.0464 | -0.1025 | -0.0496 | -0.042 | -0.0078 | -0.068 | -0.0464 | 0.0062 |
| **CIN2,3 to Cancer [HPV-16]** | 0.0206 | 0.0831 | 0.0126 | -0.0456 | -0.0005 | 0.046 | -0.0222 | 0.0085 | 0.0477 | -0.0301 | 0.0762 |
| **CIN2,3 to Cancer [HPV-18]** | 0.0722 | -0.0211 | 0.0012 | -0.0875 | 0.0218 | 0.067 | 0.0388 | -0.0076 | 0.0845 | 0.0077 | -0.144 |
| **CIN2,3 to Normal [Low-Risk HPV]** | 0.1022 | 0.067 | 0.072 | 0.0189 | 0.0716 | 0.0406 | 0.038 | -0.0105 | 0.043 | -0.0036 | -0.0392 |
| **CIN2,3 to Normal [Other High-Risk HPV]** | 0.0589 | 0.0435 | 0.0926 | 0.0045 | 0.0258 | 0.0663 | 0.0657 | 0.0515 | 0.0062 | 0.0474 | 0.0271 |
| **CIN2,3 to Normal [HPV-16]** | 0.0158 | -0.1496 | 0.0301 | 0.0136 | -0.0842 | -0.0103 | -0.0315 | 0.0116 | 0.092 | 0.0457 | -0.1156 |
| **CIN2,3 to Normal [HPV-18]** | 0.0017 | 0.073 | -0.0194 | -0.0361 | 0.0106 | 0.0144 | 0.0157 | 0.002 | -0.0121 | -0.0722 | 0.0511 |
| **CIN1 to Normal [Low-Risk HPV]** | -0.0888 | -0.0989 | -0.1264 | 0.1171 | 0.0077 | -0.003 | 0.0035 | 0.4311* | 0.037 | -0.0059 | -0.0027 |
| **CIN1 to Normal [Other High-Risk HPV]** | -0.0784 | -0.0099 | -0.0274 | -0.1099 | 0.0847 | -0.0638 | -0.0212 | 0.0281 | 0.5366* | 0.1078 | 0.0313 |
| **CIN1 to Normal [HPV-16]** | -0.0676 | -0.0763 | -0.025 | 0.0202 | -0.0622 | -0.022 | -0.0428 | 0.0518 | 0.0652 | 0.2345* | 0.3776* |
| **CIN1 to Normal [HPV-18]** | -0.0676 | -0.0763 | -0.025 | 0.0202 | -0.0622 | -0.022 | -0.0428 | 0.0518 | 0.0652 | 0.2345* | 0.3776* |
| **HPV Infection to Normal [Low-Risk HPV]** | -0.0096 | -0.0553 | 0.0034 | -0.0322 | -0.1354 | -0.0176 | -0.0531 | -0.0108 | 0.0572 | -0.0289 | 0.0641 |
| **HPV Infection to Normal [Other High-Risk HPV]** | -0.2559* | -0.1693 | -0.1174 | -0.0367 | 0.0995 | 0.0501 | 0.1084 | 0.0372 | 0.0158 | -0.1661 | -0.0498 |
| **HPV Infection to Normal [HPV-16]** | -0.1706 | -0.2534* | -0.1006 | -0.0291 | 0.1628 | 0.1183 | 0.1189 | 0.0103 | -0.2768* | 0.2111* | 0.1887* |
| **HPV Infection to Normal [HPV-18]** | -0.1706 | -0.2534* | -0.1006 | -0.0291 | 0.1628 | 0.1183 | 0.1189 | 0.0103 | -0.2768* | 0.2111* | 0.1887* |

**Appendix Table 4B.** Pairwise Correlation of Calibrated Model Inputs

|  | **HPV Infection to CIN2,3 [Low-Risk HPV]** | **HPV Infection to CIN2,3 [Other High-Risk HPV]** | **HPV Infection to CIN2,3 [HPV-16]** | **HPV Infection to CIN2,3 [HPV-18]** | **CIN1 to CIN2,3 [Low-Risk HPV]** | **CIN1 to CIN2,3 [Other High-Risk HPV]** | **CIN1 to CIN2,3 [HPV-16]** | **CIN1 to CIN2,3 [HPV-18]** | **CIN2,3 to Cancer [Other High-Risk HPV]** | **CIN2,3 to Cancer [HPV-16]** | **CIN2,3 to Cancer [HPV-18]** |
| --- | --- | --- | --- | --- | --- | --- | --- | --- | --- | --- | --- |
| **HPV Infection to CIN2,3 [Low-Risk HPV]** | 1 |  |  |  |  |  |  |  |  |  |  |
| **HPV Infection to CIN2,3 [Other High-Risk HPV]** | -0.0488 | 1 |  |  |  |  |  |  |  |  |  |
| **HPV Infection to CIN2,3 [HPV-16]** | 0.0286 | 0.0289 | 1 |  |  |  |  |  |  |  |  |
| **HPV Infection to CIN2,3 [HPV-18]** | -0.0773 | 0.0268 | 0.0593 | 1 |  |  |  |  |  |  |  |
| **CIN1 to CIN2,3 [Low-Risk HPV]** | -0.0084 | -0.0122 | 0.0018 | -0.0129 | 1 |  |  |  |  |  |  |
| **CIN1 to CIN2,3 [Other High-Risk HPV]** | 0.0291 | -0.2146* | 0.0256 | -0.0384 | -0.0907 | 1 |  |  |  |  |  |
| **CIN1 to CIN2,3 [HPV-16]** | -0.0052 | -0.0267 | -0.15 | -0.1062 | 0.0006 | 0.0455 | 1 |  |  |  |  |
| **CIN1 to CIN2,3 [HPV-18]** | 0.0707 | 0.0155 | 0.0214 | -0.1375 | 0.0959 | 0.0484 | 0.018 | 1 |  |  |  |
| **CIN2,3 to Cancer [Other High-Risk HPV]** | 0.0763 | 0.0128 | 0.0375 | 0.0088 | 0.0234 | -0.1362 | 0.0565 | 0.0113 | 1 |  |  |
| **CIN2,3 to Cancer [HPV-16]** | 0.0052 | 0.0838 | -0.0727 | 0.0663 | 0.0917 | 0.1008 | -0.0179 | -0.0513 | -0.0389 | 1 |  |
| **CIN2,3 to Cancer [HPV-18]** | -0.0162 | 0.0211 | 0.04 | 0.0179 | 0.0099 | -0.0829 | -0.0478 | -0.0263 | 0.0161 | 0.0285 | 1 |
| **CIN2,3 to Normal [Low-Risk HPV]** | -0.0001 | 0.0416 | -0.0294 | -0.0343 | 0.1301 | -0.0014 | -0.0182 | -0.0263 | -0.0101 | -0.0322 | 0.1284 |
| **CIN2,3 to Normal [Other High-Risk HPV]** | 0.0687 | 0.1234 | 0.0636 | -0.001 | 0.0349 | 0.3016* | 0.0195 | 0.0396 | 0.0136 | 0.0142 | -0.0336 |
| **CIN2,3 to Normal [HPV-16]** | -0.0403 | 0.1076 | 0.1536 | 0.0105 | -0.0384 | -0.0195 | 0.1829* | 0.0112 | 0.0556 | 0.004 | 0.0508 |
| **CIN2,3 to Normal [HPV-18]** | 0.0606 | 0.023 | -0.0224 | -0.0792 | -0.0043 | -0.0046 | 0.0067 | 0.1849* | -0.0172 | 0.0133 | 0.0694 |
| **CIN1 to Normal [Low-Risk HPV]** | -0.0471 | 0.0334 | 0.0329 | -0.0006 | 0.1335 | -0.1465 | -0.0471 | 0.0535 | -0.0872 | -0.0091 | -0.0079 |
| **CIN1 to Normal [Other High-Risk HPV]** | 0.0095 | 0.0117 | 0.0828 | 0.0292 | 0.0605 | 0.0027 | -0.0334 | 0.0199 | -0.0418 | 0.0611 | 0.077 |
| **CIN1 to Normal [HPV-16]** | 0.0425 | 0.0807 | 0.0265 | -0.0123 | 0.0499 | -0.0024 | -0.0479 | -0.0386 | -0.0298 | 0.024 | -0.1166 |
| **CIN1 to Normal [HPV-18]** | 0.0425 | 0.0807 | 0.0265 | -0.0123 | 0.0499 | -0.0024 | -0.0479 | -0.0386 | -0.0298 | 0.024 | -0.1166 |
| **HPV Infection to Normal [Low-Risk HPV]** | 0.0071 | 0.0478 | -0.0368 | -0.0517 | -0.0255 | 0.0332 | -0.0823 | -0.0515 | -0.0095 | 0.0054 | -0.0529 |
| **HPV Infection to Normal [Other High-Risk HPV]** | 0.018 | 0.0878 | -0.045 | 0.0307 | -0.0365 | -0.0377 | -0.0734 | -0.021 | 0.0113 | -0.0332 | -0.0595 |
| **HPV Infection to Normal [HPV-16]** | -0.001 | -0.091 | 0.2004* | -0.0598 | 0.0392 | -0.0469 | 0.1758* | 0.1759* | 0.1112 | 0.0243 | 0.0909 |
| **HPV Infection to Normal [HPV-18]** | -0.001 | -0.091 | 0.2004* | -0.0598 | 0.0392 | -0.0469 | 0.1758* | 0.1759* | 0.1112 | 0.0243 | 0.0909 |

**Appendix Table 4C.** Pairwise Correlation of Calibrated Model Inputs

|  | **CIN2,3 to Normal [Low-Risk HPV]** | **CIN2,3 to Normal [Other High-Risk HPV]** | **CIN2,3 to Normal [HPV-16]** | **CIN2,3 to Normal [HPV-18]** | **CIN1 to Normal [Low-Risk HPV]** | **CIN1 to Normal [Other High-Risk HPV]** | **CIN1 to Normal [HPV-16]** | **CIN1 to Normal [HPV-18]** | **HPV Infection to Normal [Low-Risk HPV]** | **HPV Infection to Normal [Other High-Risk HPV]** | **HPV Infection to Normal [HPV-16]** | **HPV Infection to Normal [HPV-18]** |
| --- | --- | --- | --- | --- | --- | --- | --- | --- | --- | --- | --- | --- |
| **CIN2,3 to Normal [Low-Risk HPV]** | 1 |  |  |  |  |  |  |  |  |  |  |  |
| **CIN2,3 to Normal [Other High-Risk HPV]** | -0.0918 | 1 |  |  |  |  |  |  |  |  |  |  |
| **CIN2,3 to Normal [HPV-16]** | 0.0076 | 0.0229 | 1 |  |  |  |  |  |  |  |  |  |
| **CIN2,3 to Normal [HPV-18]** | 0.0005 | 0.0394 | -0.0354 | 1 |  |  |  |  |  |  |  |  |
| **CIN1 to Normal [Low-Risk HPV]** | -0.0235 | 0.0127 | -0.0316 | 0.0136 | 1 |  |  |  |  |  |  |  |
| **CIN1 to Normal [Other High-Risk HPV]** | -0.0542 | 0.0658 | 0.0045 | -0.0607 | -0.0215 | 1 |  |  |  |  |  |  |
| **CIN1 to Normal [HPV-16]** | -0.0606 | 0.0279 | -0.011 | -0.0494 | 0.0525 | 0.1118 | 1 |  |  |  |  |  |
| **CIN1 to Normal [HPV-18]** | -0.0606 | 0.0279 | -0.011 | -0.0494 | 0.0525 | 0.1118 | 1.0000* | 1 |  |  |  |  |
| **HPV Infection to Normal [Low-Risk HPV]** | -0.0442 | -0.0241 | -0.0361 | -0.041 | -0.2976* | -0.0492 | 0.018 | 0.018 | 1 |  |  |  |
| **HPV Infection to Normal [Other High-Risk HPV]** | 0.0243 | -0.1584 | 0.0288 | 0.0968 | 0.0135 | -0.1426 | -0.0065 | -0.0065 | 0.0035 | 1 |  |  |
| **HPV Infection to Normal [HPV-16]** | -0.0419 | 0.0187 | -0.072 | -0.0487 | 0.0415 | 0.0653 | -0.2566* | -0.2566* | -0.037 | -0.0107 | 1 |  |
| **HPV Infection to Normal [HPV-18]** | -0.0419 | 0.0187 | -0.072 | -0.0487 | 0.0415 | 0.0653 | -0.2566* | -0.2566* | -0.037 | -0.0107 | 1.0000* | 1 |

**Model Evaluation**

We evaluated the performance of the model in terms of face-validity and external consistency by comparing model output to data from several large U.S. screening studies not used in the parameterization or calibration of the natural history model. These included the Portland Kaiser Permanente and the ASCUS/LSIL Triage Study for Cervical Cancer (ALTS) studies as well as data from the National Cancer Institute’s Surveillance Epidemiology and End Results (SEER) Program [41,98,99]. For all evaluation targets, we derived both point estimates and 95% confidence intervals from the empirical data. Baseline data from the Portland Kaiser Permanente study was used to calculate the cross-sectional, age-specific prevalence of high-risk HPV and of cervical cytology results that were high-grade squamous intraepithelial lesion or worse (HSIL+). Baseline data from the ALTS study was used to calculate the cross-sectional proportions of HPV-positive persons with CIN1 and CIN2+ (e.g., histology of CIN2 or worse) having HPV-16, HPV-18, or other high-risk HPV types. Data from SEER for 2003 were used to derive incidence of age-specific detected invasive cervical cancer in the presence of cervical cancer screening, and analogous estimates were calculated from IARC data for the U.S. prior to the widespread screening. We estimated the incidence reductions due to screening by subtracting SEER incidence rates from IARC rates by age. SEER data from 1996 to 2000 was used to calculate the proportion of invasive cervical cancer cases detected at each SEER historical stage (local, regional, and distant).

To compare model results to our evaluation targets, we simulated five screening scenarios for each of the 50 parameter sets from the calibrated natural history model. These included no screening and screening using cervical cytology at four levels of intensity: every 1, 2, 3, or 5 years from age 18 to 70. Further details about screening are presented below.

Model outputs for these simulations were based on cohorts of 1,000,000 women. Because data from these studies were derived from women with different past patterns of screening, we combined modeled outputs from our five screening scenarios to produce modeled outputs that would be consistent with a cohort of women whose cervical cancer screening patterns matched nationally observed, age-specific patterns of screening [100,101]. For the ALTS targets, age-specific modeled outputs were then collapsed across age categories using a weighted average based upon the age structure of the ALTS data. Since SEER stage at cancer detection targets were based on women with detected cervical cancer, matched modeled outputs (e.g., the number of cervical cancer cases detected at each stage for each screening scenario) were combined using screening patterns derived from case-control studies of women with diagnosed cervical cancer [102-104]. We evaluated model performance for face validity and external consistency using a benchmark of overlap between the model range and the study’s confidence interval or range. Similarity in age patterns between modeled and study outcomes based on simple visual inspection was used as a further criterion for assessing model performance. Ranges of model outcomes are derived by repeating each set of simulations with 50 good-fitting parameter sets identified via calibration.

**Modeled Cancer Prevention Strategies**

The model is capable of simulating many different screening and vaccination strategies. First we describe the subset of strategies relevant to the present analysis. In a subsequent section we provide a more general overview of the full range of prevention strategies that may be simulated with the model.

For the selected analyses presented in this article there were two main goals. The first was to evaluate the model by comparing its output in the presence of screening to several, large studies of populations undergoing cervical cancer screening. The second was to assess the impact of parameter uncertainty on choices relating to screening strategies recommened under current guidelines in the absence and presence of HPV vaccination. A complete description of screening and vaccination strategies that the model is currently capable of simulating appears later in the document.

While a comparison of all policy-relevant strategies is beyond the scope of this analysis, it is important to illustrate the relationship between capturing parameter uncertainty via calibration and quantifying this uncertainty in terms of the uncertainty of policy-relevant outcomes. For this purpose, we compare vaccination, screening, and a combination of vaccination and screening based on their model-projected cancer incidence reductions compared to no screening and no vaccination for each of the 50 randomly resampled, good-fitting parameter sets. The screening strategy chosen for this analysis is based on current U.S. screening guidelines as published by organizations such as the American Cancer Society (ACS) and U.S. Preventive Services Task Force (USPSTF). The screening strategy is used under 5 scenarios: no screening and screening every 1, 2, 3, or 5 years from ages 18 to 70. The screening strategy uses cervical cytology as a primary screening test and uses HPV triage for samples that have a cytology result of atypical squamous cells of unknown significance (ASC-US), reserving treatment of precancer for women with colposcopically and histologically confirmed CIN2 or worse. Screening test characteristics are based on review of the published literature, as presented in tables for cytology and HPV DNA testing appearing below. Based on the published literature, vaccination is assumed to be 100% protective against HPV-16 and HPV-18 for women vaccinated prior to being infected with these HPV types, and the vaccine is adminstered at age 12 prior to sexual debut.

**Appendix Table 5.** Screening and Vaccination Assumptions

|  | **Assumption** |
| --- | --- |
|  |  |
| Screening Algorithm |  |
|  |  |
| Eligibility for Screening |  |
| Starting criteria | 18 years old or 3 years after sexual debut |
| Stopping criteria (for women with abnormalities) | 70 years old |
|  |  |
| Management of Abnormal Results† |  |
| ASC-US/HPV-negative | Regular Screening |
| ASC-US/HPV-positive | Colposcopy/Biopsy |
| LSIL | Colposcopy/Biopsy |
| HSIL | Colposcopy/Biopsy |
| Treatment Threshold | Biopsy-confirmed CIN2+ |
|  |  |
| Intensive Follow-up ‡ |  |
| Screening interval | 6-12 months |
| Continued until | 3 consecutive normal results |
|  |  |
| Vaccination |  |
|  |  |
| Age at vaccination | Prior to 12 and before sexual debut |
| Efficacy for adolescents prior to sexual activity | 100% |
| Immunity Duration | Lifelong |

| * | ASC-US: Atypical Squamous Cells of Unknown Significance; HPV: Human Papillomavirus; LSIL: Low-grade Squamous Intraepithelial Lesion; HSIL: High-grade Squamous Intraepithelial Lesion; CIN: Cervical Intraepithelial Neoplasia; DNA: Deoxyribonucleic Acid |
| --- | --- |
| † | Samples that are determined to be have an ASC-US cytology result are tested with a reflex HPV DNA test (Hybrid Capture II) to triage who is sent for colposcopy and biopsy |
| ‡ | For all women referred to colposcopy regardless of colposcopy and biopsy results or treatment |

## Model Capabilities: All Prevention Strategies

While the previous section described a strategy involving cytology screening with HPV DNA testing reserved to triage ASC-US results, the model has the capability of simulating a variety of screening strategies. In this section we offer a more general overview of the full range of strategies that may be simulated with the model, including further details on dimensions relevant to decision analytic modeling of prevention strategies, omitted from the selected analyses presented in the main article.

In the model, each screening strategy is defined by the primary screening test, option for triage of abnormal results, the ages at which screening begins and ends, the frequency of screening, and the intensity of follow-up screening for abnormal results. **Appendix Figure 13** summarizes the structure of a more comprehensive analysis of screening strategies for the U.S. reflecting this broad array of dimensions. These strategies can be refined by the population level of adherence to the screening strategy as well as the adherence to diagnostic follow-up and treatment. Screening strategies can be combined such that the assigned strategy switches at a given age. The two screening tests currently incorporated into the model are cervical cytology and the HPV DNA test. The tests can be used alone or in combination. The model is not limited to these two tests, however, and hypothetical tests can also be modeled to assess investments in new technology. The frequency of primary screening can be specified either as a fixed frequency for the entire cohort of simulated women or as a distribution of frequencies across the cohort. If the latter, each woman is randomly assigned a screening frequency in accordance to the specified distribution. The screening schedules are defined by the start age and the interval in months between primary screens. Following a primary screening test, a secondary or “triage” test may be specified for designated abnormal results. This triage test can be performed using the same sample as that collected for use in the primary test but alternative approaches can be assessed. We assume that colposcopy occurs in the same month as screen detection. More frequent screening can be specified for a period of time subsequent to an abnormal screening result. The frequency of this intensive screening is assumed to be every 12 months but can be made more or less frequent. In concordance with current guidelines, we have specified that a woman must receive three sequential negative tests before returning to her routine screening schedule.

**Appendix Figure 13.** Decision Tree for U.S. Analysis

**Appendix Figures 14 Panels A, B, and C** show schematics of test results leading to diagnostic work-up and treatment as well as intensive follow-up for modeled screening strategies.

**Appendix Figure 14.** Schematics of Modeled Screening Strategies

** ASC-US = atypical squamous cells of unknown significance.*

*† LSIL = low-grade squamous intraepithelial lesions; HSIL = high-grade squamous intraepithelial lesions.*

*‡ Treatment follows standard guidelines.*

***Schematics of Modeled Strategies.*** Three screening strategies are shown in the three panels. In Panel A, a strategy using Cervical Cytology with triage HPV DNA testing for ASC-US is shown. Women who test normal on cytology return to routine screening. Women who have ASC-US results on cytology but are negative for high-risk HPV types, are screened more frequently until they have 3 consecutive negative test results. Women who have LSIL or worse cytology results and women with ASC-US results who test positive for high-risk HPV types are referred for diagnostic work-up using colposcopy and biopsy. Women with invasive cancer are referred for staging and appropriate management. Women with CIN2,3 are referred for treatment and post-treatment follow-up. All other women are referred for more frequent screening until they have 3 consecutive negative test results. In Panel B, HPV DNA testing with Cervical Cytology triage for high-risk HPV positive results is shown. Panel C shows Cervical Cytology and HPV DNA testing used in combination. For Panel B and Panel C, referal for colposcopy and biopsy as well as treatment and follow-up conform to rules consistent with those depicted in Panel A.

## *Screening Test Characteristics*

The test characteristics of cervical cytology are defined in terms of the presence of true CIN. The model provides the facility for age-specific (ages 0-18, 18-35, 35-50, 50+) differences in sensitivity and specificity. The specificity (probability of a negative cytology test given the absence of CIN, regardless of HPV infection status) is assumed to be 95%. The probabilities that the cytology test is positive with the presence of CIN1 and CIN2,3 are assumed to be 70% and 80% respectively. These test characteristics were derived from previously published studies and verified against more recent studies of test performance [4,7,105-107]. Because cytology is not a binary test, positive cytology test results are subdivided conditional on true CIN status (including women with no CIN who have positive cytology results due to false positive test results) [4,7]. **Appendix Table 6** shows the probability of different cytology results conditional on underlying CIN status and testing positive on the test. For example, a women with true CIN1 who tests positive has a 46.8% chance of having an ASC-US results and a 3.5% change of having an HSIL result.

**Appendix Table 6.** Probability of cytology result given true health state

|  | **Normal** | **CIN1** | **CIN2,3** | **Cancer** |
| --- | --- | --- | --- | --- |
| **ASC-US** | 0.659 | 0.468 | 0.265 | 0.286 |
| **ACIN23** | 0.045 | 0.049 | 0.115 | 0.000 |
| **LSIL** | 0.269 | 0.448 | 0.450 | 0.286 |
| **HSIL** | 0.027 | 0.035 | 0.170 | 0.428 |

In contrast to cervical cytology, HPV DNA test characteristics are defined as the probability of the test being positive conditional on the presence or absence of high-risk (oncogenic) HPV DNA types detected by Hybrid Capture 2 (Digene Corporation, Gaithersburg, MD). Most large scale screening studies conducted in real-world situations report the sensitivity of HPV DNA tests in terms of the detection of CIN2,3+ [105,108-111]. Because the prevalence of each CIN category as well as high-risk HPV DNA change by age and the HPV type distribution within each CIN category also changes by age, test characteristics defined in terms of the presence of CIN2,3+ are likely to change by age as well. In our model, the sensitivity of an HPV DNA test for CIN is an output. We verified the face validity of our test characteristic assumptions by comparing the implied sensitivity of an HPV DNA test conducted on previously unscreened model cohorts of various ages to those reported in large studies of screening tests (**Appendix Table 7**).

**Appendix Table 7** Mapping of Modeled HPV DNA Test Characteristics: Presence/Absence of High Risk HPV DNA to Test Characteristics in Terms of Presence/Absence of CIN2,3+ By Age and Assumed Sensitivity and Specificity for High Risk HPV DNA (Means and Ranges)

|  | **HPV DNA Test Characteristics in Terms of Probability of Detecting the Presence/Absence of High Risk HPV (Sensitivity, Specificity)** | | | | | | |
| --- | --- | --- | --- | --- | --- | --- | --- |
|  | **(100, 100)** | **(92.5, 100)** | **(100, 92.5)** | **(92.5, 92.5)** | **(100, 85)** | **(85, 100)** | **(85, 85)** |
| **Sensitivity for CIN2,3+** |  |  |  |  |  |  |  |
| **Age** |  |  |  |  |  |  |  |
| 30 | 89.2  [79.0 - 97.0] | 82.5  [74.0 - 89.0] | 89.7  [81.0 - 97.0] | 83.5  [75.0 - 90.0] | 90.7  [83.0 - 97.0] | 75.7  [68.0 - 82.0] | 77.3  [71.0 - 83.0] |
| 40 | 83.0  [72.0 - 94.0] | 76.7  [67.0 - 87.0] | 84.3  [74.0 - 95.0] | 78.2  [69.0 - 88.0] | 85.5  [76.0 - 95.0] | 70.5  [61.0 - 80.0] | 73.0  [65.0 - 81.0] |
| 50 | 79.5  [66.0 - 93.0] | 73.5  [61.0 - 86.0] | 80.8  [68.0 - 93.0] | 74.8  [63.0 - 86.0] | 82.5  [71.0 - 94.0] | 67.5  [56.0 - 79.0] | 70.7  [61.0 - 80.0] |
| 60 | 79.5  [65.0 - 93.0] | 73.3  [60.0 - 86.0] | 81.2  [68.0 - 94.0] | 75.2  [63.0 - 87.0] | 82.3  [70.0 - 94.0] | 67.3  [55.0 - 79.0] | 70.7  [61.0 - 80.0] |
|  |  |  |  |  |  |  |  |
|  |  |  |  |  |  |  |  |
| **Specificity for CIN2,3+** |  |  |  |  |  |  |  |
| **Age** |  |  |  |  |  |  |  |
| 30 | 91.2  [89.0 - 93.0] | 91.8  [90.0 - 93.0] | 84.2  [82.0 - 86.0] | 85.0  [83.0 - 86.0] | 77.3  [75.0 - 79.0] | 92.3  [90.0 - 94.0] | 79.0  [77.0 - 80.0] |
| 40 | 93.5  [92.0 - 96.0] | 94.2  [93.0 - 96.0] | 86.3  [85.0 - 88.0] | 87.2  [86.0 - 89.0] | 79.5  [78.0 - 81.0] | 94.5  [93.0 - 96.0] | 80.7  [80.0 - 82.0] |
| 50 | 94.2  [93.0 - 96.0] | 94.3  [93.0 - 96.0] | 87.2  [86.0 - 89.0] | 87.2  [86.0 - 89.0] | 79.8  [79.0 - 81.0] | 94.8  [94.0 - 96.0] | 80.8  [80.0 - 82.0] |
| 60 | 94.8  [93.0 - 96.0] | 95.3  [94.0 - 97.0] | 87.7  [86.0 - 89.0] | 88.2  [87.0 - 89.0] | 80.7  [79.0 - 82.0] | 95.7  [94.0 - 97.0] | 81.2  [80.0 - 82.0] |

### *Main Vaccine Parameters*

HPV vaccines are modeled as protecting against HPV-16 and HPV-18. The model allows the flexibility of changing vaccine coverage; how many doses are given and what age they are given; and the probability of provoking an immune response and level of immune response provoked. Vaccine coverage is the percentage of the population given the vaccine. Since the full vaccination course involves 3 doses, the model allows for the possibility of some covered individuals receiving only 1 or 2 doses instead of the full 3-dose course. We have operationalized the efficacy of the vaccine by accounting for both the probability of evoking a vaccine-induced immune response and the strength of that immune response. One parameter, termed “vaccine factor” refers to the conditional probability of the vaccine producing an immune response in an individual if that individual has received vaccine doses. A second parameter is the “vaccine degree” and refers to the percent reduction in the type-specific probability of being infected with a vaccinated HPV type conditional upon being vaccinated and having had an vaccine-induced immune response invoked (e.g., a vaccinated woman who is capable of having an immune response to the vaccine has her age-specific probability of being infected with HPV-16 reduced by a certain percentage). The model also allows for vaccine efficacy to depend on partial vaccination. Additionally, age at vaccination is modeled as a cumulative distribution of the probability of being vaccinated by that age. Vaccination can happen at one fixed age by shifting the cumulative distribution from 0 to 1 at a given age.

## Health-Related Quality of Life

Quality of life weights are incorporated into the model enabling population statistics such as the quality-adjusted life expectancy. Age-specific quality of life weights from nationally representative U.S. EQ-5D utility scores were specified for women without detected cervical cancer [112]. **Appendix Table 8** shows the age-specific quality of life weights for women without invasive cervical cancer. These were adjusted for women with detected cancer using stage-specific quality of life weights [7]. The quality of life adjustments (assumed to be multiplicative) for diagnosed invasive cervical cancer were Local, 0.68; Regional, 0.56; and Distant, 0.48. Health-related quality of life was not further adjusted for the presence of HPV infection or CIN. Within the model, optional decrements to quality of life can be specified for screening events including participation in a screening test and participation in colposcopy.

**Appendix Table 8.** Age-Specific Quality of Life Weights for Women without Cervical Cancer

| Age (years) | Quality of Life |
| --- | --- |
| 11–19 | 1 |
| 20–29 | 0.913 |
| 30–39 | 0.893 |
| 40–49 | 0.863 |
| 50–59 | 0.837 |
| 60–69 | 0.811 |
| 70–79 | 0.771 |
| 80+ | 0.724 |
